# MicroRNA-34a: Potent Tumor Suppressor, Cancer Stem Cell Inhibitor, and Potential Anticancer Therapeutic

**DOI:** 10.3389/fcell.2021.640587

**Published:** 2021-03-08

**Authors:** Wen (Jess) Li, Yunfei Wang, Ruifang Liu, Andrea L. Kasinski, Haifa Shen, Frank J. Slack, Dean G. Tang

**Affiliations:** ^1^Department of Pharmacology and Therapeutics, Roswell Park Comprehensive Cancer Center, Buffalo, NY, United States; ^2^Experimental Therapeutics Graduate Program, Roswell Park Comprehensive Cancer Center, Buffalo, NY, United States; ^3^Department of Gynecology, Affiliated Hospital of Jining Medical University, Jining, China; ^4^Department of Biological Sciences, Purdue University, West Lafayette, IN, United States; ^5^Department of Nanomedicine, Houston Methodist Research Institute, Weill Cornell Medical College, Houston, TX, United States; ^6^Department of Pathology, Beth Israel Deaconess Medical Center, Harvard Medical School, Boston, MA, United States

**Keywords:** miR-34a, microRNA, cancer stem cells, miRNA therapeutics, cancer cell heterogeneity

## Abstract

Overwhelming evidence indicates that virtually all treatment-naive tumors contain a subpopulation of cancer cells that possess some stem cell traits and properties and are operationally defined as cancer cell stem cells (CSCs). CSCs manifest inherent heterogeneity in that they may exist in an epithelial and proliferative state or a mesenchymal non-proliferative and invasive state. Spontaneous tumor progression, therapeutic treatments, and (epi)genetic mutations may also induce plasticity in non-CSCs and reprogram them into stem-like cancer cells. Intrinsic cancer cell heterogeneity and induced cancer cell plasticity, constantly and dynamically, generate a pool of CSC subpopulations with varying levels of epigenomic stability and stemness. Despite the dynamic and transient nature of CSCs, they play fundamental roles in mediating therapy resistance and tumor relapse. It is now clear that the stemness of CSCs is coordinately regulated by genetic factors and epigenetic mechanisms. Here, in this perspective, we first provide a brief updated overview of CSCs. We then focus on microRNA-34a (miR-34a), a tumor-suppressive microRNA (miRNA) devoid in many CSCs and advanced tumors. Being a member of the miR-34 family, miR-34a was identified as a p53 target in 2007. It is a bona fide tumor suppressor, and its expression is dysregulated and downregulated in various human cancers. By targeting stemness factors such as NOTCH, MYC, BCL-2, and CD44, miR-34a epigenetically and negatively regulates the functional properties of CSCs. We shall briefly discuss potential reasons behind the failure of the first-in-class clinical trial of MRX34, a liposomal miR-34a mimic. Finally, we offer several clinical settings where miR-34a can potentially be deployed to therapeutically target CSCs and advanced, therapy-resistant, and p53-mutant tumors in order to overcome therapy resistance and curb tumor relapse.

## Introduction

Most adult tissues and organs harbor adult stem cells (SCs) responsible for tissue homeostasis and regeneration. Upon division, SCs can give rise to transit-amplifying (TA) cells, which will terminally differentiate into functional cells. Adult SCs are long lived and may generate the progeny throughout life (Batlle and Clevers, 2017). Tissues such as the intestinal epithelium and the hematopoietic system continuously self-renew through SCs for rapid expansion and tissue repair (Doulatov et al., 2012; Clevers, 2013). Overwhelming evidence has demonstrated the presence of a relatively small fraction of cancer cells, cancer stem cells (CSCs), with stem-like properties in most early-stage and treatment-naive human malignancies. These CSCs possess the capability of self-renewal and tumor initiation and propagation and are more tolerant of standard treatment modalities, such as chemotherapy and radiation therapy. Therefore, CSCs are postulated to be responsible for tumor maintenance, therapy resistance, relapse, and metastasis.

MicroRNAs (miRNAs) are ∼22-nucleotide (nt) short non-protein coding RNAs that are key posttranscriptional regulators of gene expression (Bartel, 2004). Mammalian genomes are estimated to encode as many as 1,000 unique miRNAs. It is estimated that 60% of human protein coding genes are subject to regulation by miRNAs (Friedman et al., 2009). miRNAs contain a seed region (nucleotides 2–8) that binds to partially complimentary sequences in the 3′-untranslated regions (3′-UTR) of their target messenger RNAs (mRNA) for gene silencing through either suppression of translation or mRNA degradation. miRNAs are universally dysregulated in almost all solid and hematological malignancies, implicating their involvement in tumorigenesis (Garzon et al., 2010; Ling et al., 2013; Lionetti et al., 2013). In the context of cancer, miRNAs can be functionally classified into two types: oncogenic miRNAs (OncomiRs) and tumor suppressor miRNAs.

**FIGURE 1:**
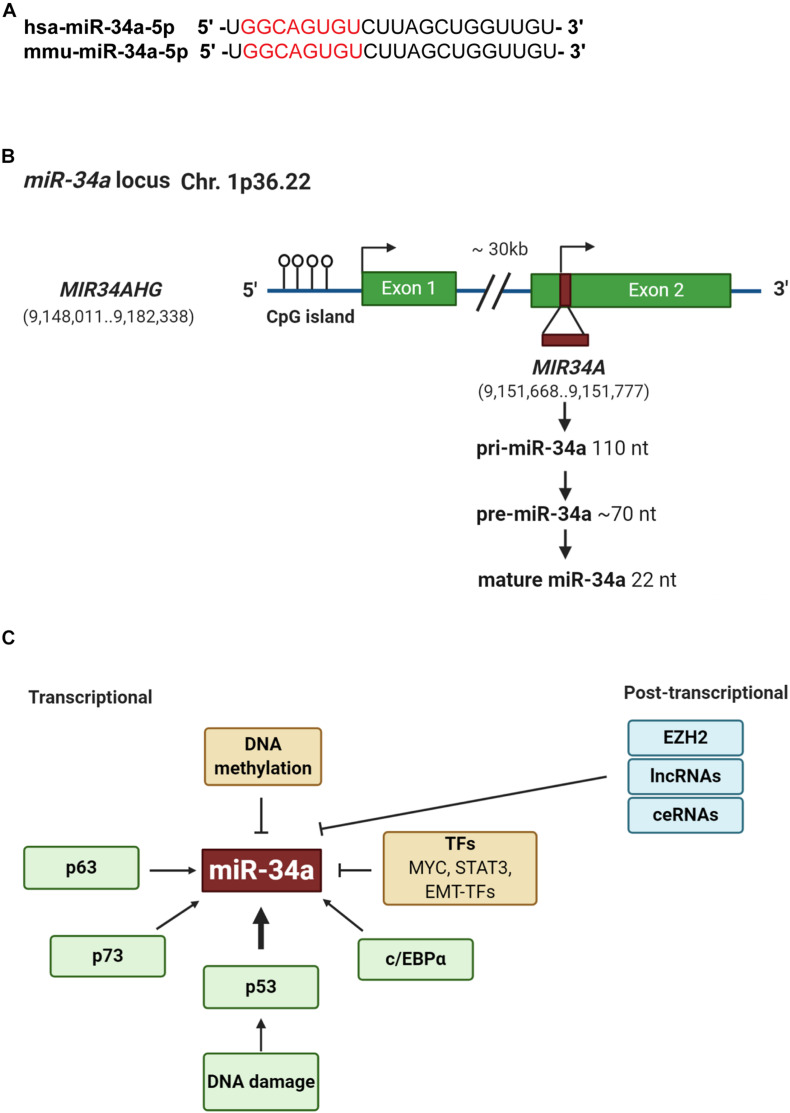
Genomic structure and regulation of miR-34a. **(A)** Sequence alignment of the mature human and mouse miR-34a. The seed sequences are highlighted in red. **(B)** Structure of miR-34a genomic loci. Green boxes represent exons. Horizontal arrow marks transcription start site. **(C)** Regulation of miR-34a. At the transcriptional level, p53, p63, and p73 induces whereas DNA methylation, TME-TFs such as SNAIL and ZEB1, STAT3, and Myc repress miR-34a expression. At the posttranscriptional level, EZH2, lncRNAs, and competing endogenous RNAs (ceRNAs) may negatively regulate miR-34a expression.

**FIGURE 2:**
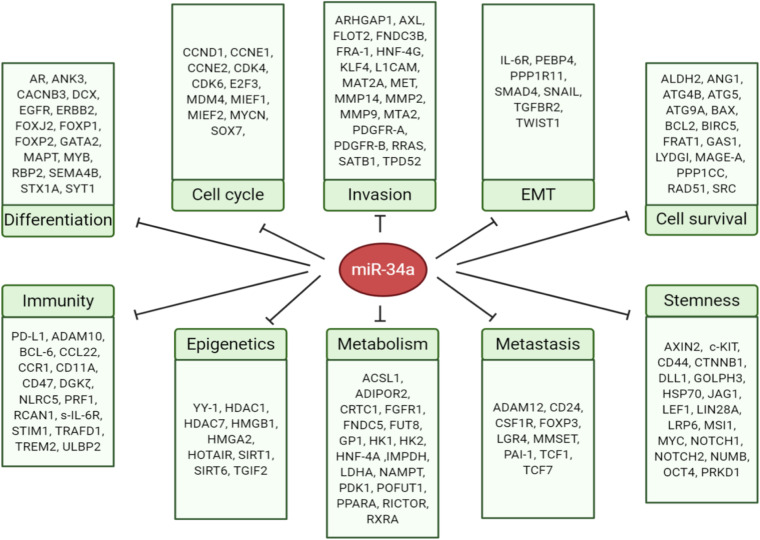
MicroRNA-34 regulates cancer-relevant cellular processes by targeting key factors. The direct targets of miR-34a are grouped into 10 cellular processes according to their main functions.

The miR-34 family, along with the let-7 and miR-200 families, are three major tumor-suppressive miRNA families. The miR-34 family consists of three members: miR-34a (Figure 1), miR-34b, and miR-34c. miR-34a is encoded in the second exon of a gene located on chromosome 1p36.22 (Figure 1B), whereas miR-34b and miR-34c are expressed from a polycistronic transcript encoded on chromosome 11q23.1. The mature miR-34a sequence consists of 22 nucleotides and shares 86% (19/22 nt) and 82% (18/22 nt) homology with miR-34b and miR-34c, respectively (Bader, 2012), suggesting that the miR-34 family members could share a similar set of targets and thus be functionally redundant. miR-34a is the prevailing family member in normal human tissues, whereas miR-34b/c are expressed at lower levels in most tissues except the lung, ovary, testes, and trachea (Rokavec et al., 2014). miR-34a in both human and mouse have identical seed sequences, i.e., 5′-GGCAGUGU-3′ (Figure 1A). Loss of miR-34a expression occurs in a wide range of solid tumors and hematological malignancies (Bader, 2012). Since miR-34 was identified as a p53 target in 2007 (Figure 1C), extensive research has demonstrated miR-34a as an essential mediator of p53 functions and a potent tumor suppressor. Indeed, miR-34a suppresses tumor growth and cancer progression by inhibiting multiple tumor-promoting processes including the cell cycle, epithelial-to-mesenchymal transition (EMT), metastasis, stemness, and tumor immunity and by inducing tumor-inhibitory events such as apoptosis and senescence (Figure 2; see discussions below). miR-34a regulates these cellular processes by downregulating target mRNAs, and to date, more than 200 miR-34a targets have been reported and/or validated (Supplementary Table 1).

As CSCs act as the “seed” of the tumor with quiescent and self-renewal properties, they are likely responsible for therapy resistance and recurrence that are key challenges in cancer therapy. Notably, miR-34a is a potent suppressor of CSCs in various cancers. For instance, we previously found miR-34a to be devoid in prostate cancer stem cell (PCSC) populations (e.g., those identified as CD44^+/hi^), and overexpression of miR-34a induced pronounced inhibitory effects on tumor growth and metastasis *in vivo* by negatively regulating PCSCs (Liu et al., 2011). Below, we further discuss the role of miR-34a in regulating CSCs in a spectrum of cancers, especially with respect to regulating the factors involved in tumor microenvironment (TME), tumor immune microenvironment (TiME), metabolism, and EMT. We shall also discuss the recent advances in translational research of miR-34a and offer several clinical settings where miR-34a could be deployed to therapeutically target CSCs for tackling therapy resistance and tumor relapse.

## Regulation of miR-34a Expression

In addition to p53-regulated expression, miR-34a can also be regulated in a p53-independent manner (Figure 1C). Indeed, miR-34a has been shown to be transcriptionally regulated via TP63, TP73, and other transcription factors (TFs) such as STAT3, MYC, and EMT-TFs, as well as posttranscriptionally regulated by various long non-coding RNAs (lncRNAs) (Slabáková et al., 2017; Figure 1C). In addition, miR-34a expression can be context dependent and may dynamically change during EMT, hypoxia, and inflammation (Siemens et al., 2011; Yang et al., 2012; Li H. et al., 2017). miR-34a expression is also regulated through epigenetic regulation. For example, enhancer of zeste homolog 2 [EZH2; a histone 3 lysine 27 (H3K27) methyltransferase], in conjunction with HOTAIR, has been shown to silence miR-34a by induction of heterochromatin (Li C.H. et al., 2017). Moreover, Sirt7, a NAD^+^-dependent class III histone deacetylase, binds to the promoter of miR-34a and deacetylates the H3K18ac, thus repressing miR-34a expression (Zhang et al., 2015).

Many lncRNAs also epigenetically regulate miR-34a expression (Figure 1C). For example, the lncRNA HOTAIR represses miR-34a by enhancing DNA methylation of the *mir-34a* promoter via recruiting and binding to polycomb repressive complex 2 (PRC2), which promotes tumor metastasis by controlling EMT-related genes (Liu et al., 2015). In addition, lincRNA regulator of reprogramming (ROR) negatively regulates miR-34a expression by inhibiting histone H3 acetylation in the *mir-34a* promoter, leading to the reversal of gemcitabine-induced autophagy and apoptosis in breast cancer (Chen Y.M. et al., 2016). The lncRNA myocardial infarction associated transcript (MIAT) can interact with DNMT3a and epigenetically silence miR-34a expression by hypermethylating the *mir-34a* promoter (Fu et al., 2018). Interestingly, a novel lncRNA, Lnc34a, has been reported to recruit Dnmt3a via PHB2 and HDAC1 to methylate and deacetylate the *mir-34a* promoter simultaneously, hence epigenetically silencing miR-34a expression independently of p53 (Wang et al., 2016). Lnc34a promotes CSC self-renewal, and Lnc34a asymmetry leads to cell fate asymmetry in CSC division by directly targeting miR-34a to cause its spatial imbalance (Wang et al., 2016). Moreover, Lnc34a may promote bone metastasis through suppressing miR-34a, which leads to inhibition of Smad4 and alterations of transcription of the downstream genes [i.e., connective tissue growth factor (CTGF) and interleukin (IL)-11] that are associated with bone metastasis (Zhang et al., 2019).

## An Evolving View of the CSC Concept

**FIGURE 3:**
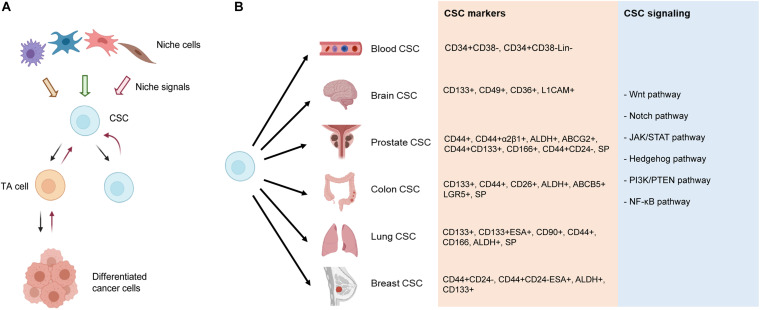
Current understanding of CSCs and CSC markers in representative tumors. **(A)** CSC “hierarchy.” In early-stage treatment-naive tumors, the CSC, residing in the CSC niche consisting of many different niche cells, undergoes asymmetric cell division (ACD) generating one CSC and one transient amplifying (TA) cell, with the latter dividing rapidly and developing into phenotypically “differentiated” cancer cells (indicated by forward black arrows) that may well represent the bulk of the tumor. Upon therapeutic interventions and/or (epi)genetic mutations (not shown), the TA cell and even fully differentiated cancer cells can be reprogrammed (i.e., dedifferentiate) into CSCs, a property often called plasticity. **(B)** Reported CSC markers in the representative cancers. Shown on the right are several major developmental and signaling pathways commonly deregulated in CSCs and various cancer types. SP, side population.

Heterogeneity is an omnipresent feature of cancer cells *in vitro* and *in vivo*, which manifest distinct phenotypic characteristics, epigenetic states, and functional attributes (Tang, 2012). Within this tumor heterogeneity are CSCs, which generally represent a small subpopulation of the bulk tumor cells in early-stage treatment-naive tumors. CSCs (Figure 3) are operationally defined as the stem-like cancer cells that possess some or most of the normal SC properties such as relative quiescence but with great proliferative potential, the ability to self-renew and differentiate, and, importantly, the capability to regenerate and long-term propagate tumors (Tang, 2012; Plaks et al., 2015). Using the above definition, CSCs, presumed to reside in special tumor microenvironment, i.e., the CSC niches (Figure 3A), are found to be yet heterogeneous in that different subsets of CSCs can be enriched using different techniques and/or different sets of phenotypic markers (Figure 3B; see below). Significantly, cancer cells also display phenotypic and functional plasticity (Tang, 2012) in that TA cells, or even differentiated cancer cells, may, under the therapeutic pressure and/or due to genetic/epigenetic alterations, undergo dedifferentiation (reprogramming) to revert to stem-like cancer cells (Figure 3A). Due to our much advanced understanding of the adult hematopoietic SCs (HSCs), CSCs were first reported in human acute myeloid leukemia (AML), in which leukemic cells expressing the same markers as normal adult HSCs (CD34^+^CD38^–^) were much more efficient at engrafting and propagating the leukemia in immune-deficient mice (Lapidot et al., 1994; Uckun et al., 1995; Doulatov et al., 2012). Breast cancer was the first human solid tumor that was shown to contain CSCs, which displayed the CD44^+^CD24^–/lo^ phenotype (Al-Hajj et al., 2003). CSCs, with various phenotypic properties, have subsequently been reported in many different malignancies (Figure 3B) including, among others, cancers of the brain (Singh et al., 2004), colon (Dalerba et al., 2007; O’Brien et al., 2007; Ricci-Vitiani et al., 2007), and prostate (Collins et al., 2005; Huss, 2005; Patrawala et al., 2005, 2006).

CSCs are commonly identified and enriched using diverse strategies including flow cytometry-based sorting using cell surface markers, e.g., CD44 and CD133, and functional approaches, including side population (SP) analysis, Aldefluor assay, and sphere formation coupled with serial sphere passaging (Tang, 2012). Like normal SCs, CSCs in the same type of cancer could be phenotypically and functionally heterogeneous. Indeed, a variety of CSC markers have been reported in human cancers (Figure 3B). CD34^+^CD38^–^ is the most widely used CSC marker in leukemia (Lapidot et al., 1994; Uckun et al., 1995; Doulatov et al., 2012), whereas CD133^+^ was described as the first brain CSC marker (Singh et al., 2004). Since this early finding, many other markers for brain CSCs have also been reported including integrin alpha 6, CD36, and L1CAM (Desai et al., 2019). Similarly, diverse markers have been reported for PCSCs including SP, CD44^+^, CD44^+^α2β1^+^, ABCG2^+^, CD44^+^CD133^+^, ALDH^+^, CD166^+^, and CD44^+^CD24^–^ (Skvortsov et al., 2018; Figure 3B). Colon CSCs were originally identified using CD133^+^ marker (Ricci-Vitiani et al., 2007). Since then, reports have identified CD44^+^ cells to be enriched for CSC-like properties, CD26^+^ cells capable of initiating tumor formation and facilitating EMT, and LGR5^+^ normal intestinal SCs serving as the tumor cells of origin (Desai et al., 2019). Reported CSC markers in lung cancer include CD133^+^, CD44^+^, CD166^+^, CD90^+^, ALDH^+^, and SP phenotype (Zakaria et al., 2017). In addition, lung CSCs have been isolated by using the combination of CD133 with other markers such as ABCG2, CXCR4, and epithelial-specific antigen (ESA) (Bertolini et al., 2009). In breast cancer, CD44^+^CD24^–^, ALDH^+^, and CD133^+^ are by far commonly used molecular markers to enrich and characterize breast CSC (BCSC) (Ginestier et al., 2007; Fillmore and Kuperwasser, 2008; Wright et al., 2008; Gupta et al., 2009). Many markers have been reported to correlate with the SC properties in breast cancer, including enhanced PKH26 dye retention, low proteasome activity, and high expression of molecules like CD29, CD61, CD49f, PROCR, Sca-1, MUC1, Thy1, vimentin, osteonectin, CK18, and GATA3 (Yang et al., 2017). CSCs in different cancers are commonly deregulated in critical developmental and signaling pathways including Wnt, Notch, Janus kinase/signal transducers and activators of transcription (JAK/STAT), Hedgehog, phosphatidylinositol-3-kinase (PI3K)/phosphatase and tensin homolog (PTEN), and nuclear factor kappa B (NF-κB) (Matsui, 2016; Figure 3B).

The canonical hierarchical CSC model is established upon our knowledge of the cellular hierarchy in normal HSCs and hematopoiesis. The key features comprise the following: (1) a substantial fraction of cellular heterogeneity observed in tumors results from its hierarchical organization, which is often, but not always, reminiscent of the hierarchy in the tissue of origin; (2) tumor cell hierarchies are fueled by CSCs, which are rare and typically quiescent, whereas the bulk of the tumor is composed of non-CSCs, which are capable only of transient proliferation and therefore do not contribute to long-term growth; (3) CSC identity is hardwired, meaning there is limited plasticity in the tumor hierarchy; and (4) CSCs are resistant to standard chemotherapy and radiation, which preferentially target non-CSCs explaining relapse after treatment (Batlle and Clevers, 2017). On the other hand, not all solid tumors fit into the “rigid” hierarchical HSC hierarchy model (Clevers, 2015). Recent studies using lineage-tracing and cell-ablation strategies have shed novel insights into the CSC concept (Figure 3A). For example, CSCs may not necessarily be rare and quiescent depending on the tumor stages and on whether tumors have been treated or not. Indeed, even normal adult SCs in the intestinal crypts can be quite abundant in their niche and are generally dormant or slow cycling but can proliferate vigorously throughout the life (Barker et al., 2007). Importantly, both SCs and CSCs are instructed by niche signals following neutral competition dynamics (Batlle and Clevers, 2017). The mode of SC division can be either asymmetrical (i.e., generating a differentiated daughter cell and another SC) or symmetrical (generating two SCs or two differentiated daughters), as evidenced in the epidermis (Doupé et al., 2010), stomach (Leushacke et al., 2013), and intestinal crypts (Lopez-Garcia et al., 2010; Snippert et al., 2010). Furthermore, the CSC “hierarchy” could be plastic in that TA cells and even fully differentiated cells could dedifferentiate to more immature cells in response to the niche signals (Figure 3A). For example, in a study in which stem cell-, basal-, or luminal-like cells were isolated from breast cancer cell cultures, all three subpopulations were able to generate the cells of the other two phenotypes given certain conditions (Gupta et al., 2011). Thus, CSCs and the non-CSC state may not be hardwired, and the phenotypic transition reflects the adaptation to particular environmental cues.

## miR-34a Regulation of CSCs

### Blood Cancer

Blood cancer encompasses three main types: leukemia, lymphoma, and multiple myeloma (MM). miR-34a plays a vital role in regulating hematopoiesis. For example, miR-34a perturbs B-cell development by targeting Foxp1 (a known B-cell oncogene) and inducing a block at the pro-B-cell to pre-B-cell transition, leading to a reduction in mature B cells (Rao et al., 2010). Intriguingly, several studies have reported that miR-34a is functionally lost in chronic lymphocytic leukemia (CLL) but overexpressed in AML (Isken et al., 2008; Mraz et al., 2009; Zenz et al., 2009), suggesting that miR-34a expression may affect the phenotype of leukemia cells in various ways.

Furthermore, miR-34a promotes megakaryocyte (MK) differentiation and inhibits proliferation in erythroleukemia by directly targeting the transcription factor MYB, an important regulator of cell differentiation at multiple decision forks in hematopoiesis (Navarro et al., 2009). miR-34a overexpression in primary human CD34^+^ hematopoietic precursors significantly increased MK colony number by approximately 30% (Navarro et al., 2009), indicating that miR-34a contributes to MK differentiation from primitive HSCs.

**FIGURE 4:**
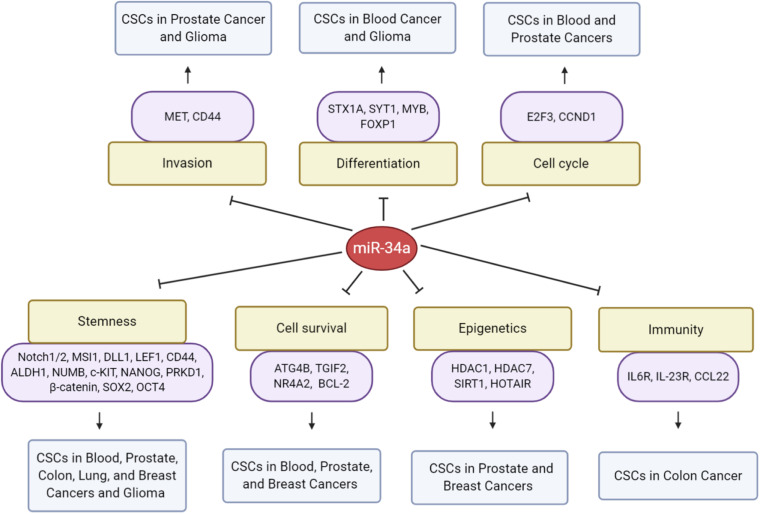
MicroRNA-34a regulation of CSCs. Presented are direct targets of miR-34a and biological consequences on CSCs in the indicated cancer types.

MicroRNA-34a has major functions in myeloid differentiation as well, and manipulation of miR-34a levels can re-establish myeloid differentiation in primary AML blast cells with CEBPA mutations (Pulikkan et al., 2010). The transcription factor, CCAAT enhancer binding protein alpha (C/EBPα), is one of the major regulators in granulopoiesis and is downregulated by various mechanisms in AML (Schuster and Porse, 2006). Mechanistically, C/EBPα directly transactivates miR-34a by interacting with the *mir-34a* genomic region (Figure 1C), and miR-34a, in turn, targets E2F3 during granulopoiesis (Pulikkan et al., 2010). miR-34a overexpression resulted in granulocytic differentiation of AML blast cells as assessed morphologically and by increased expression of myeloid markers, such as CD11b and granulocyte colony-stimulating factor receptor (G-CSFR) (Pulikkan et al., 2010). In chronic myeloid leukemia (CML), miR-34a impairs the survival of CD34^+^ hematopoietic stem/progenitor cells by targeting ATG4B (Figure 4), a core autophagy protein (Rothe et al., 2014). Moreover, forced expression of miR-34a sensitizes CD34^+^ CML cells to imatinib mesylate, a tyrosine kinase inhibitor (TKI) (Rothe et al., 2014). Consequently, a combination of miR-34a with TKIs might be more effective in targeting the leukemic stem/progenitor cells.

Multiple myeloma, a B-cell malignancy, is the second most common blood cancer characterized by the presence of clonal plasma cell proliferation and bone lesions (Corre et al., 2015). It has been shown that miR-34a overexpression inhibits MM CSC growth (Wu et al., 2016). Mechanistically, miR-34a directly targets transforming growth interaction factor 2 (TGIF2) and induces apoptosis, resulting in the inhibition of MM CD138^+^CD34^+^ CSC xenograft growth and lytic bone lesions (Wu et al., 2016).

### Brain Tumor

Brain tumors are among the most lethal of all cancers. Gliomas are the most common and deadly brain tumors in adults, and medulloblastoma (MB) is the most common brain tumor in children. Human brain tumor CSCs, also known as tumor-propagating cells (TPCs), are identified as CD133^+^CD15^+^ and possess clonogenic and multilineage differentiation capacity, as well as the ability to initiate tumors following orthotopic xenograft transplantation (Singh et al., 2004; Read et al., 2009). The Abounader research team, for the first time, studied the role of miR-34a in brain tumors with a focus on gliomas (Li et al., 2009) and found that miR-34a expression was reduced in human gliomas and that miR-34a levels in p53-mutant gliomas were lower than in p53 wild-type tumors. They also uncovered the oncogenes MET, NOTCH1, and NOTCH2 as direct targets of miR-34a in glioma stem cells (GSCs). Subsequent work from the group showed that miR-34a re-expression inhibited various protumorigenic properties of GSCs, including cell proliferation (via inducing G1/S cell cycle arrest), survival, and migration (Guessous et al., 2010). Importantly, miR-34a induced GSC differentiation and decreased GSC markers CD133 and NESTIN, while it increased astrocyte marker glial fibrillary acidic protein (GFAP) and the oligodendrocyte marker claudin-11 (Guessous et al., 2010). These data suggest that miR-34a reduces glioma stemness and promotes cell differentiation.

Msi1 is an evolutionarily conserved RNA-binding protein considered to play roles in maintaining the stemness of neural stem/progenitor cells in adults. It is highly expressed in multiple tumor types, such as glioma, MB, and cervical cancer (Toda et al., 2001; Yokota et al., 2004; Ye et al., 2008). Msi1 knockdown cells showed ∼50% reduction in glioma sphere formation when compared to control, indicating that Msi1 is a functional regulator of GSCs (Vo et al., 2011). Interestingly, miR-34a negatively regulated GSC stemness by targeting Msi1 (Vo et al., 2011). In human MB, miR-34a expression reduced the percentage of CD15^+^CD133^+^ MB TPCs, inhibited their stemness properties, and promoted neural differentiation in MB by targeting NESTIN and Notch ligand Delta-like 1 (DLL1) (de Antonellis et al., 2011).

The above-mentioned studies indicate that miR-34a potently inhibits brain CSC growth by targeting multiple pathways (Figure 4) and, as such, might serve as a brain tumor therapeutic target. In support, through a targeted delivery system, miR-34a has been demonstrated to efficiently accumulate in glioma TPCs, inhibit their stemness and chemoresistance, and prolong the survival of TPC-bearing mice (Jiang et al., 2020). miR-34a delivered via tailored vehicles reduced SOX2 expression by 35% and also inhibited the clonogenicity of glioma TPCs (Jiang et al., 2020).

### Prostate Cancer

Prostate cancer (PCa) is the most common cancer among men and the second leading cause of cancer-related deaths in the United States. PCSCs with high tumor-initiating and metastatic capacities are enriched in the side population (Patrawala et al., 2005), CD44^+^ (Patrawala et al., 2006), and CD44^+^α2β1^+^ (Patrawala et al., 2007) subpopulations. Our group was the first to identify the role of miR-34a in regulating PCSCs and PCa metastasis (Liu et al., 2011). miR-34a was found to be underexpressed in several above-mentioned PCSC populations, especially the CD44^+/hi^ PCa cells, and overexpression of miR-34a in bulk PCa cells or purified CD44^+^ cells elicited pronounced inhibitory effects on tumor growth and metastasis *in vivo*. By contrast, introduction of miR-34a antagomirs in CD44^–^ PCa cells promoted tumor regeneration and metastasis. Importantly, systemic delivery of miR-34a inhibited metastasis and prolonged the survival of tumor-bearing animals (Liu et al., 2011). Mechanistically, miR-34a suppressed PCSC properties (e.g., prostasphere establishment, migration and invasiveness of CD44^+^ cells, and serial tumor transplantation) by directly targeting CD44 (Figure 4). This study (Liu et al., 2011) demonstrated that miR-34a acts as a crucial negative regulator of (CD44^+/hi^) PCSCs and establishes a strong rationale for developing miR-34a as a novel therapeutic against aggressive and metastatic PCa (Liu et al., 2011). Interestingly, underexpression of miR-34a in CD44^+^ PCa cells might not be related to p53 expression or activity (Liu et al., 2012), suggesting p53-independent regulation of miR-34a. Cheng et al. were the first to investigate the role of miR-34 in regulating normal prostate SCs, and their work showed that combined inactivation of p53 and miR-34a in mouse prostate epithelium led to expansion of prostate SC compartment as well as development of high-grade prostatic intraepithelial neoplasia and early invasive adenocarcinoma (Cheng et al., 2014). Consistent with *in vivo* observations, inactivation of both miR-34 and p53 led to accelerated EMT-dependent growth, enhanced self-renewal capacity, and increased cell motility in prostate SCs derived from the proximal region of prostatic ducts (Cheng et al., 2014).

Interestingly, recent preclinical studies have shown that a chalcone derivative, rubone, acts as a miR-34a modulator to elicit anti-CSC efficacy in PCa. Rubone was first reported to upregulate the intracellular miR-34a in hepatocellular carcinoma, resulting in significant tumor shrinkage (Xiao et al., 2014). Micellar delivery of rubone reversed chemoresistance, inhibited tumor cell migration and invasion, and decreased the CSC population of paclitaxel-resistant PCa cells in a p53-independent pathway (Wen et al., 2017). Rubone promoted the effect of paclitaxel through upregulating miR-34a and downregulating the levels of miR-34a targets E-cadherin, SIRT1, and cyclin D1 as well as aldehyde dehydrogenase (ALDH) activity, which is a CSC marker (Wen et al., 2017). Another study also revealed that treatment of taxane-resistant PCa cell lines with rubone micelles decreased the ALDH^*hi*^ CSC subpopulation in a dose-dependent manner, suggesting that miR-34a can reverse the chemoresistance of PCSCs (Lin et al., 2019). Codelivery of rubone and docetaxel is expected to synergistically and efficiently inhibit tumor growth by killing both CSCs and bulk tumor cells (Lin et al., 2019).

### Colorectal Cancer

Colon CSCs or CCSCs are thought to arise from, or at least share common properties with, normal colon SCs (Clevers, 2011). As in normal SCs, disruption of asymmetric division can alter the balance between self-renewal and differentiation in CSCs and impact tumor growth (Cicalese et al., 2009; Sugiarto et al., 2011). Bu et al. (2013) was the first to identify a role for the Notch-targeting miR-34a in controlling asymmetric division of CCSCs (Figure 4). Similar to normal colon SCs, CCSCs in early-stage, well-differentiated tumors can undergo either self-renewing symmetric division (producing two daughter CCSCs) or asymmetric division (producing a CCSC and a differentiated, non-CCSC cell), and this decision was controlled by the miR-34a level (Bu et al., 2013). miR-34a, a cell-fate determinant of CCSCs, acts as a toggle switch for Notch signaling that dictates binary symmetric vs. asymmetric fates in early-stage dividing CCSCs: whereas high miR-34a levels decreased both symmetric and asymmetric division resulting in fewer CCSCs and more non-CCSC daughter cells, low miR-34a levels increased symmetric CCSC–CCSC division while at the same time decreased asymmetric division resulting in more CCSC daughter cells and fewer non-CCSC daughter cells (Bu et al., 2013). Interestingly, miR-34a levels have to be at a “sweet spot” in the middle for asymmetric division in this system, since either too much or too little miR-34a abolishes asymmetric division (Bu et al., 2013). These data reveal the key role of miR-34a in fine-tuning the balance between asymmetric and symmetric CSC divisions.

Numb is a canonical cell fate determinant for various CSCs and has been used as a marker for distinguishing symmetric vs. asymmetric division (O’Brien et al., 2012). Bu and his colleagues from the same research group further elucidated the relationship between the microRNA cell fate determinant miR-34a and the canonical protein cell fate determinant Numb. They found that miR-34a and Numb synergize to regulate self-renewal vs. differentiation of early stage CCSCs by forming an incoherent feedforward loop, since miR-34 targets NUMB and Notch1, whereas Notch1 is inhibited by NUMB protein in a converging pathway (Bu et al., 2016). Importantly, during simulated inflammation provoked by tumor necrosis factor α (TNF-α), loss of miR-34a resulted in SCs shifting to symmetric division leading to an increase in intestinal SCs, suggesting that miR-34a provides a safeguard mechanism against SC proliferation induced by inflammation or oncogenic mutation (Bu et al., 2016). Thus, there is a possibility that loss of miR-34a may lead to increased number of CCSCs and cancer progression, considering that inflammation is one of the hallmarks during cancer development.

Inflammation generally plays a protective role in inducing regeneration to repair the tissue damage. However, chronic inflammation can be a major risk of cancer and may lead to tumorigenesis over time. It has been demonstrated that miR-34a may act as a central safeguard to protect the inflammatory SC niche and regulate reparative regeneration for inflammation-induced colon oncogenesis (Wang et al., 2018). miR-34a deficiency led to colon tumorigenesis after *C. rodentium* (a mouse mucosal pathogen) infection, where Th17 (T helper 17) cell infiltration in close proximity to colon SCs was observed (Wang et al., 2018). During the proinflammatory response, miR-34a suppressed Th17 cell differentiation and expansion by targeting the IL-6 receptor (IL-6R) and IL-23R, hindered Th17 cell recruitment to colon epithelium by targeting chemokine CCL22, and inhibited IL-17 induced SC proliferation by targeting an orphan receptor, IL-17RD (Wang et al., 2018). These data indicate that enhancing miR-34a levels may have additional benefits of unleashing tumor immune responses to combat cancer cells by repressing Th17 cells and IL-17 stimulation of CSCs in the TME.

Colon cancers cells with an impairment in DICER1, a major protein in miRNA biogenesis, manifest increased features of tumor stemness and EMT, which are associated with miR-34a downregulation (Iliou et al., 2014). Another group also showed that miR-34a suppresses CRC stemness, inhibits migration and invasion, and improves chemosensitivity by targeting c-Kit (Siemens et al., 2013). c-Kit, also known as CD117 or stem cell factor (SCF), is a type III receptor tyrosine kinase involved in the regulation of HSCs and stemness in ovarian cancer (Kent et al., 2008; Chau et al., 2013).

The expression of miR-34 family has also been shown to be epigenetically silenced by CpG methylation in CRC cell lines and in primary CRC (Figure 4). Jiang and Hermeking uncovered the *in vivo* functions of *miR-34a/b/c* in suppression of intestinal tumorigenesis (Jiang and Hermeking, 2017). Combined deletion of *miR-34a/b/c* increased the number of intestinal SCs in association with deregulated Notch and Wnt signaling (Jiang and Hermeking, 2017). *miR-34a/b/c*-deficient adenomas showed elevated proliferation and decreased apoptosis and displayed pronounced bacterial infiltration, which may be associated with the decreased infiltrating immune cells and downregulation of barrier proteins (Jiang and Hermeking, 2017).

### Lung Cancer

Lung cancer is the most lethal malignancy worldwide with a high metastasis and recurrence rate. Many studies have shown that both small-cell (SCLC) and non-small cell (NSCLC) lung cancers contain CSCs (Dovey et al., 2008; Eramo et al., 2008; Shi et al., 2014). As in many other tumors, lung CSCs have been enriched and purified using cell surface markers such as CD133, CD34, CD90, and CD44 (Tang, 2012). Low levels of miR-34a were detected in highly tumorigenic CD44^*hi*^ cells from lung cancer patients presenting malignant pleural effusion, and miR-34a replacement inhibited colony formation of CD44^*hi*^ cells (Basak et al., 2013), suggesting that miR-34a negatively regulates the stemness of NSCLC CSCs. In support of this suggestion, our group also reported miR-34a-mediated inhibition of tumor-initiating capacity of the CD44^*hi*^ lung CSCs (Shi et al., 2014). In the study, restoration of miR-34a expression, in three NSCLC cell lines that possess different p53 status, inhibited both clonal and clonogenic properties (Shi et al., 2014). This suggests that the tumor-inhibitory effects of miR-34a could be p53 independent. miR-34a expression specifically in CD44^*hi*^ H460 cells greatly inhibited the tumor-regenerating activity, whereas miR-34a antagonists promoted tumor regeneration in CD44^*lo*^ H460 cells (Shi et al., 2014). These data are consistent with CD44 being a direct and functional target of miR-34a in PCSCs (Liu et al., 2011; Figure 4) and help establish a strong rationale for developing miR-34a as a novel therapeutic agent against NSCLC.

In fact, a systemic delivery system of miR-34a packaged in lipid nanoparticles has been reported to specifically target lung CSCs (Shi et al., 2013). The nanovector-delivered miR-34a greatly inhibited the ability of B16F10-CD44^+^ CSC-like cells to form spheroids *in vitro* as well as tumor growth in mice by attenuating CD44 expression (Shi et al., 2013).

### Breast Cancer

Breast cancer is a heterogeneous disease with multiple tumor subtypes. miR-34a is downregulated in breast cancer and is negatively correlated with an aggressive phenotype (Kang et al., 2015). Expression of miR-34a occurs upon luminal commitment and differentiation and serves to inhibit the expansion of the pool of Numb^+^CD49f^+^ mammary stem cells and early progenitor cells by modulating the Wnt/β–catenin signaling pathway, likely in a p53-independent fashion (Bonetti et al., 2019). In other words, miR-34a has a dual role in regulating cell proliferation and commitment toward luminal differentiation in mammary progenitor cells. miR-34a plays similar functions in breast cancer, as chronic expression of miR-34a in triple-negative mesenchymal-like breast cancer (enriched in CSCs) could promote luminal-like differentiation, restrict the CSC pool and inhibit tumor propagation (Bonetti et al., 2019).

Breast cancer stem cells (BCSCs) were the first to be prospectively identified among solid tumors (Al-Hajj et al., 2003). Identification of BCSCs from tumor samples and breast cancer cells relies mainly on CD44^+^/CD24^–^ or ALDH phenotypes (Ginestier et al., 2007; Fillmore and Kuperwasser, 2008; Gupta et al., 2009). miR-34a suppresses BCSC properties by directly targeting functionally important signaling molecules (Figure 4) including Notch1 (Park et al., 2014; Kang et al., 2015), SIRT1 (Ma et al., 2015), PRKD1 (Protein kinase D1) (Kim Do et al., 2016), and C22ORF28 (Lin et al., 2017). miR-34a inhibits breast cancer stemness and increases the chemosensitivity to doxorubicin and paclitaxel partially by downregulating the Notch1 pathway, with reduced CD44^+^/CD24^–^ BCSCs and CSC marker ALDH1 (Park et al., 2014; Kang et al., 2015). In addition, miR-34a inhibits mammosphere formation and proliferative potential of BCSCs *in vitro* and *in vivo*, at least partially by downregulating SIRT1, ALDH1, and Nanog (Ma et al., 2015). PRKD1, a direct target of miR-34a, was capable of regulating cancer stemness in chemoresistant human breast cancer cells by altering GSK3/β-catenin signaling, and ectopic miR-34a expression reduced PRKD1 resulting in suppressed self-renewal of BCSCs (Kim Do et al., 2016).

In addition to Notch1, miR-34a may also negatively regulate the aggressiveness and chemoresistance of BCSCs by targeting key epigenetic modulators such as HDAC1/HDAC7 (Wu et al., 2014). Of interest, miR-34a has been reported to sensitize BCSCs to the killing by natural killer (NK) cells via inducing the expression, on CSCs, of NK cell ligands (Zhang et al., 2016). Specifically, miR-34a promotes the expression in BCSCs of activating NK cell ligands such as H60, Rae1, CD155, and CD112, while it inhibits the expression of inhibitory receptors including H-2K, H-2D, and Qa-1 (Zhang et al., 2016). In addition, miR-34a, delivered via a non-viral plasmid system, significantly inhibited the tumor-initiating properties of long-term-cultured BCSCs *in vivo* (Lin et al., 2017).

### Other Cancers

In addition to the widely studied cancers discussed above, miR-34a has also been implicated to restrict the biological and tumorigenic properties of CSCs in other human cancers, which we summarize in Supplementary Table 2.

## miR-34a Regulation of Immune and Stromal Cells in the CSC Niche

Like normal SCs that reside in a protected niche, CSCs are also localized in a special tumor microenvironment called the CSC niche, which might be anatomically distinct (e.g., more hypoxic and more acidic) from the overall TME (Hanahan and Lisa, 2012). Conceivably, non-CSC tumor cells, which often represent the bulk population in untreated tumors, may constitute a critical part of the CSC niche. During tumor progression to a more malignant state, the CSC and non-CSC state may also dynamically evolve under the changing influence of the TME in general and the CSC niche in particular (Fessler et al., 2013). Multiple immune and stromal (mesenchymal) cell types within the CSC niche produce factors such as cytokines that may stimulate CSC self-renewal, induce angiogenesis, and further recruit immune and other stromal cells, which form a vicious cycle that promotes tumor cell invasion and metastasis by secreting additional factors (Plaks et al., 2015). Studies in the past decade have implicated miR-34a as a critical modulator of the CSC niche.

### Infiltrated Immune Cells

The role of the CSC niche in modulating the level of tumor immunity is of great interest. TP53 mutations in myelodysplastic syndromes and secondary AML patients confer an immunosuppressive phenotype (Sallman et al., 2020). Those TP53 mutant patients display significantly reduced numbers of cytotoxic T lymphocytes (CTLs) and helper T cells, as well as decreased NK cells with increased regulatory T cells (Treg cells) and myeloid-derived suppressor cells (MDSCs) (Sallman et al., 2020). Notably, downregulation of miR-34a was observed in TP53 mutant patients (Sallman et al., 2020), suggesting a close link between miR-34a expression levels, p53 mutation status, and tumor immunity.

#### Natural Killer Cells

Natural Killer cells, the effector lymphocytes of the innate immune system, play pivotal functions in cancer immune surveillance. Malignant cells express ligands for the NK cell immunoreceptor NKG2D, which mediates early recognition and elimination by CTLs and provides an innate barrier against tumor development (Champsaur and Lanier, 2010). ULBP2, one of the NKG2D ligands (NKG2DL), has been detected on the surface of tumor cells and identified as a prognostic biomarker in different tumors (Paschen et al., 2009; Nückel et al., 2010). miR-34a functions as a repressor of ULBP2 by directly targeting its 3′-UTR (Figure 2), resulting in the repression of NKG2D-mediated immune surveillance in melanoma and enhanced tumor progression (Heinemann et al., 2012). In contrast, miR-34a can promote the cytotoxic susceptibility of BCSCs to NK cells by inducing overexpression of ligands for the NK cell-activated receptors in BCSCs (Zhang et al., 2016). The contrasting effects observed in melanoma vs. breast cancer suggest that miR-34a may regulate NK cell activity (i.e., inhibitory vs. activating) in tumor- and context-dependent manners.

#### Tumor-Associated Macrophages

Tumor-associated macrophages (TAMs) are key orchestrators of TME in general and TiME in particular, directly affecting tumor growth, angiogenesis, and extracellular matrix remodeling (Solinas et al., 2010). TAMs derived from peripheral blood monocytes are recruited to the TME and can be polarized into M1 or M2 macrophages depending on the cytokine milieu. M1 macrophages highly express inducible nitric oxide synthase (iNOS) and TNF-α and mediate proinflammatory and immune responses that inhibit oncogenic processes, whereas M2 macrophages express Arginase 1 (ARG1) and drive anti-inflammatory responses, tumor progression, angiogenesis, and immunosuppression. miR-34a may regulate the macrophage polarization in the TME, as miR-34a expression in triple negative breast cancer (TNBC) cells induces THP-1 monocytes differentiation into M1-like CD86-positive macrophages (Weng Y.S. et al., 2019). Conversely, miR-34a suppresses the polarization of pan-macrophages (F4/80) and M2-like macrophages evidenced by decreases in CD163 and CD206 (Weng Y.S. et al., 2019). On the one hand, this study indicates that miR-34a may elicit antitumorigenic immune responses and tumor-suppressive functions by inducing M1 macrophage polarization. On the other hand, decreased miR-34a may upregulate the activation of JAK2/STAT3 pathway leading to increased IL-6, which in turn can promote the M2 macrophage polarization, contributing to doxorubicin chemoresistance in uterine leiomyosarcoma (Zhang et al., 2020).

Clinical use of programmed cell death-1 (PD-1) checkpoint inhibitors is frequently associated with cardiac toxicity due to proinflammatory reactions. Interestingly, inhibition of miR-34a has been shown to suppress M1 macrophage polarization via targeting KLF4, a critical regulator of macrophage M1/M2 polarization, and improve the cardiac functions impaired by PD-1 inhibitor (Xia et al., 2020).

#### Myeloid-Derived Suppressor Cells

Myeloid-Derived Suppressor Cells (MDSCs) are a heterogeneous population of early myeloid progenitors and possess strong immunosuppressive functions including suppression of CTL and NK/T cell activities (Hanahan and Lisa, 2012). MDSCs are often elevated in infectious and inflammatory pathological conditions. By directly targeting N-Myc, miR-34a may induce the expansion of MDSCs in the bone marrow and spleen as a result of reduced apoptosis (Chen S. et al., 2016). Overexpressed miR-34a changes the cytokine expression profile in MDSCs with increased TNF-α and iNOS, thus partially attenuating lung tumor growth (Chen S. et al., 2016). These data indicate that miR-34a might skew the differentiation of MDSCs to the proinflammatory macrophage M1 phenotype.

#### T Cells

The primary biological functions of PD-1/PD-L1 are to maintain peripheral tolerance and T-cell responses within a desired physiological range. PD-1/PD-L1 signaling induces T-cell exhaustion, an important mechanism that limits T-cell activity in the presence of chronic antigen stimulation (Wei et al., 2018). However, upregulation of PD-L1 in tumors renders cancer cells invisible to CTLs leading to overall tumor immunosuppression. An inverse correlation between PD-L1 and miR-34a expression has been observed in various cancers, and miR-34a may enhance the immune recognition in cancer cells by directly targeting PD-L1, resulting in enhanced T-cell responses and an antitumor effect (Wang et al., 2015; Cortez et al., 2016; Anastasiadou et al., 2019). For example, miR-34a, delivered via liposomal nanoparticle MRX34 to lung cancer xenograft models grown in immunocompetent mice, increased tumor-infiltrating CD8^+^ T cells, while it decreased the exhausted CD8^+^PD1^+^ T cells and Treg cells (Cortez et al., 2016). Importantly, the combined use of MRX34 and radiotherapy resulted in an even greater increase in CD8^+^ T cells (Cortez et al., 2016), suggesting the improved therapeutic potential of combining miR-34a with standard therapies such as radiotherapy.

MicroRNA-34a has also been shown to suppress Th17 cell infiltration to the colon SC niche during inflammation, suggesting that miR-34a may act as a central safeguard to protect the inflammatory SC niche (Wang et al., 2018). Mechanistically, miR-34a suppressed Th17 cell recruitment, differentiation, and expansion by targeting IL-6R, IL-23R, and CCL22 and also inhibited IL-17-induced colon SC proliferation (Wang et al., 2018). Considering that infiltration of Th17 cells is predictive of poor prognosis for CRC patients (Tosolini et al., 2011), miR-34a might function as a regulator of the SC niche to combat immunosuppression.

CCL22 has been implicated in the tumorigenic process by binding to its receptor CCR4 on the surface of Treg cells, consequently recruiting the immunosuppressive cells to the TME and facilitating tumor cell escape from immune surveillance. Yang et al. demonstrated that, in hepatocellular carcinoma (HCC), miR-34a suppresses CD4^+^CD25^+^ Treg cell recruitment into the TME by directly targeting CCL22 (Yang et al., 2012). An inverse association between miR-34a expression and abundance of Treg cells was observed in HBV^+^ HCC tissues (Yang et al., 2012), highlighting that the miR-34a-CCL22 pathway regulates Treg cell recruitment into the HCC TME.

MicroRNA-34a may also modulate primary T cells. For instance, miR-34a has been shown to enhance T cell activation through repressing the expression of diacylglycerol kinase ζ (DGKζ), a protein that regulates T-cell activation after engagement of the T-cell receptor (Shin et al., 2013). Conversely, miR-34a overexpression in primary T cells led to inhibition of T-cell activation, proliferation, survival, and effector functions via modulating intracellular calcium and NF-κB signaling (Diener et al., 2018; Hart et al., 2019a). Moreover, miR-34a might act as major hub in the T-cell regulatory networks by simultaneously targeting 14 mRNAs that are crucial for T-cell development and functions (Hart et al., 2019b). These contradictory findings suggest that further analyses will be needed to dissect the true impact of miR-34a on primary human T cells.

### Cancer-Associated Fibroblasts

A myriad of factors produced by CSCs and endothelial cells in the TME can transform normal fibroblasts into cancer-associated fibroblasts (CAFs), which are present in abnormally high numbers in the CSC niche (Kalluri and Zeisberg, 2006). CAFs, in general, promote tumor growth and metastasis via cell–cell interactions and cross-talk with tumor cells by secreting growth factors, cytokines, and exosomes (Yang et al., 2019). miR-34a may be involved in cancer progression promoted by CAFs. For example, CAFs enhanced oral squamous cell carcinoma progression through transferring miR-34a-devoid exosomes to cancer cells, and, mechanistically, the miR-34a-5p/AXL axis induces EMT via the AKT/GSK-3β/β-catenin signaling pathway (Li et al., 2018). Another study showed that cancer-associated mesenchymal stem cells (MSCs), a subtype of CAFs, contribute to the EMT and hepatocarcinogenesis via lncRNA MSC-upregulated factor (MUF) interaction with ANXA2 and miR-34a (Yan et al., 2017). Particularly, MUF, significantly upregulated in HCC-MSCs, acted as a competing endogenous RNA to sponge out miR-34a leading to Snail1 upregulation and EMT activation in HCC (Yan et al., 2017). Collectively, these recent findings provide insights on miR-34a regulation of CAFs and suggest another potential therapeutic strategy of targeting tumor-promoting CAFs using miR-34a.

### Endothelial Cells

An early study showed that miR-34a may impede endothelial progenitor cell-mediated angiogenesis via inhibiting Sirt1 and inducing senescence (Zhao et al., 2010). More recent studies corroborated miR-34a suppression of tumor angiogenesis in various cancers. For example, miR-34a inhibited angiogenesis in bladder cancer, as assessed by reduced endothelial cell tube formation *in vitro* and reduced vascular endothelial growth factor (VEGF) expression and CD31^+^ vessel density within tumors (Yu et al., 2014). Similarly, miR-34a may negatively regulate tumor angiogenesis by directly targeting both VEGFA and the lncRNA TUG1 (Dong et al., 2016).

### Tumor-Associated Sensory Nerves

Nerve fibers arisen from the peripheral nervous system are an integral component in the TME of solid tumors (Ayala et al., 2008; Magnon et al., 2013). During tumor development, nerve fibers form and infiltrate tumor parenchyma, and the density of these nerves has been associated with poor clinical outcomes (Ayala et al., 2008; Magnon et al., 2013), suggesting that newly formed adrenergic nerve fibers may likely promote tumor growth. A recent study by Amit et al. revealed an intriguing molecular mechanism of cancer-nerve crosstalk in head and neck squamous cell carcinoma (HNSCC), in which loss of TP53 leads to adrenergic transdifferentiation of tumor-associated sensory nerves through loss of miR-34a (Amit et al., 2020). Shuttling of cancer-derived, miR-34a-devoid extracellular vesicles (EV) to cancer-associated neurons positively regulates EV-derived axonogenic signals, resulting in autonomic reprogramming of existing nerves that support tumor growth (Amit et al., 2020). The study implicates miR-34a in curbing adrenergic nerve activity during cancer progression and raises the possibility of employing miR-34a to target axonal growth and the adrenergic nervous system for HNSCC treatment. Whether the proposed miR-34a inhibition of tumor-nerve crosstalk is applicable to other solid tumors remains to be determined.

## miR-34a Regulation of Cancer Metabolism

Cancer cells alter their metabolism to support growth, proliferation, and long-term maintenance. In particular, cancer cells generate energy and biomaterials by flexible utilization of fuel sources and consuming modes with increased aerobic glycolysis that complements the output of (sometimes reduced) oxidative phosphorylation, known as the Warburg Effect (Hanahan and Lisa, 2012). Lactate dehydrogenase A (LDHA), a critical enzyme in glycolysis, was associated with cell proliferation, metastasis, and poor prognosis (Xiao et al., 2016). miR-34a suppresses glycolysis and cell proliferation in breast cancer through directly targeting LDHA (Xiao et al., 2016). miR-34a may also impact the function of tumor infiltrated lymphocytes (TILs) through regulating LDHA expression (Ping et al., 2018). Hypoxia-driven downregulation of miR-34a in gastric cancer led to increased LDHA expression and lactate levels in TILs and impaired function of Th1 cells and CTLs (Ping et al., 2018). The stemness gene LIN28B promotes cancer stemness and metastatic properties by enhancing glycolysis and lactate secretion, partly through modulating the MYC/miR-34a signaling pathway (Chen C. et al., 2019), suggesting that miR-34a may regulate CSCs through rewiring cancer metabolism (Figure 2).

## miR-34a and EMT

EMT refers to the process whereby epithelial cancer cells acquire a mesenchymal phenotype that facilitates their migration, invasion, and metastatic dissemination (Scheel and Weinberg, 2012). The epithelial cancer cells undergoing EMT frequently acquire self-renewing and tumor-initiating capabilities associated with CSCs, driven by activation of the EMT transcription factors or EMT-TFs (Batlle and Clevers, 2017). Notably, the expression of EMT-TFs can be transient and dynamic, which primes epithelial cancer cells toward a CSC-like state transiently, and the cells can return to the epithelial phenotype (Schmidt et al., 2015). Extensive studies have shown that EMT-TFs may repress miR-34a expression (Figure 1C) and that miR-34a may negatively regulates EMT in various cancers through targeting many key genes in several interwoven tumor biology processes (Figure 2). First, miR-34a can suppress EMT by directly targeting EMT-TFs such as SNAIL (Siemens et al., 2011), ZEB1 (Siemens et al., 2011), ZNF281 (Hahn et al., 2013), and TWIST1 (Jiang and Hermeking, 2017). In fact, SNAIL, miR-34a, and ZNF281 form a feed-forward regulatory loop to control EMT (Hahn and Hermeking, 2014). Second, miR34a may inhibit EMT through the Wnt pathway via targeting Axin2 and LEF1 (Wang et al., 2020). miR-34 increases nuclear GSK-3β leading to decreased Snail expression and EMT repression (Kim et al., 2013). Third, miR-34a has been shown to inhibit EMT by targeting Smad4 through the TGFβ/Smad pathway (Qiao et al., 2015; Huang et al., 2018). Finally, miR-34a may suppress EMT by inhibiting STAT3 signaling via targeting PPP1R11 and CSF1R (Li H. et al., 2017; Shi et al., 2020). Interestingly, restoration of miR-34a expression in pancreatic CSCs upon treatment with HDAC inhibitors results in transcriptional inhibition of SNAIL, SLUG, and ZEB1 and increased E-cadherin expression (Nalls et al., 2011), suggesting that miR-34a may be able to reverse the process of EMT.

## miR-34a Regulation of CSCs Via Epigenetic Mechanisms

SIRT1, an NAD-dependent deacetylase, deacetylates p53 and limits the ability of p53 to transactivate its target genes. SIRT1 has been reported to be a direct target of miR-34a during apoptosis (Yamakuchi et al., 2008). miR-34 may epigenetically and negatively regulate the cell cycle and apoptosis via targeting SIRT1. Mechanistically, by targeting SIRT1, miR-34a increases acetylated p53 and p53 transcriptional targets p21 and PUMA (Yamakuchi et al., 2008). Consequently, dysregulation of the miR-34a/SIRT1 axis may play a role in the self-renewal and maintenance of BCSCs (Ma et al., 2015). In support, either ectopic miR-34a expression or SIRT1 knockdown suppressed mammosphere formation in BCSCs and decreased expression of stemness makers ALDH1, BMI1, and NANOG (Ma et al., 2015). The study suggests that miR-34a may restrict CSCs through downregulating SIRT1 and subsequently inducing p53-dependent apoptosis (Figure 2).

Another epigenetic mechanism whereby miR-34a inhibits CSCs is through targeting HDAC. For example, miR-34a directly represses HDAC1 leading to induction of the cell-cycle-dependent kinase inhibitor p21 and inhibition of cancer cell proliferation independently of p53 status (Shen et al., 2013). HDAC7 has also been identified as novel target of miR-34a in breast cancer. Repression of HDAC1 and HDAC7 by miR-34a reduced deacetylation of HSP70 K246, which promotes cancer cell survival and therapy resistance by inhibiting autophagy (Wu et al., 2014). Considering the inverse association between miR-34a and HDAC1/HDAC7 in CD44^+^CD24^–^ BCSCs, miR-34a might regulate CSC functions via targeting the HDAC1/HDAC7-HSP70 K246 axis.

Yin Yang-1 (YY1), a zinc finger protein and member of the GLI-Kruppel family, is a ubiquitous transcription factor that can epigenetically regulate gene expression depending on interacting partners, promoter context, and chromatin structure (Khachigian, 2018). YY1 is identified as a direct target of miR-34a (Figure 2) and contributes to carcinogenesis in several cancers (Chen et al., 2011; Wang et al., 2014; Khachigian, 2018). In addition, YY1 can stimulate the expression of EGFR, and loss of p53 induces *EGFR* promoter activity via YY1 (Bheda et al., 2008). Interestingly, in glioma cells, miR-34a may downregulate the expression and promoter activity of *EGFR* through YY1 leading to growth inhibition (Yin et al., 2013), which suggests a novel mode of mechanism by which EGFR is modulated by the p53–miR-34a–YY1 pathway.

## ceRNA: An Interesting Concept in Understanding the miR-34a Network

The competing endogenous RNA (ceRNA) concept was first proposed by Dr. Pandolfi in 2011. It states that both protein-coding transcripts and non-coding RNAs may share multiple microRNA recognition elements (MREs) and that both can cross-talk through MREs, the letters of a “RNA language,” to compete for microRNA binding (Salmena et al., 2011). Under this concept, ceRNAs comprise protein coding transcripts, lncRNAs, transcribed pseudogenes, and circular RNAs. In the context of cancer, recent studies have shown that increased ceRNAs can promote cancer progression, stemness, metastasis, and therapy resistance via sponging out miR-34a and regulating its availability (Figure 1C), thus counteracting the tumor suppressive function of miR-34a (Supplementary Table 3). The first two reported ceRNAs of miR-34a are TUG1 lncRNA and XIST lncRNA (Dong et al., 2016; Song et al., 2016), which function as miR-34a sponges and promote tumorigenesis and cancer progression by increasing cell proliferation and angiogenesis.

### The Protein-Coding Genes as miR-34a ceRNAs

There are ∼20,000 protein-coding genes in the human genome, many of which are identified to harbor numerous MREs. The transcript of c-Myc, a well-known oncogene, can upregulate CD44 expression by functioning as a ceRNA to sponge miR-34a, which subsequently leads to suppression of cell growth and invasion capability and repression of chemoresistance (Weng J. et al., 2019). Since CD44 is a CSC regulator in many cancers and is a direct target of miR-34a (e.g., Liu et al., 2011), it implies that both c-Myc and CD44 could act as ceRNAs for miR-34a. As indirect support, a positive correlation between c-Myc and CD44 expression was found in samples from urothelial carcinoma patients (Weng J. et al., 2019). CD44 may also function as a ceRNA to enhance NK-cell-mediated cytotoxicity to liver CSCs by regulating ULBP2 (Weng J. et al., 2019). As ULBP2 is a target of miR-34a, CD44 competes the binding of miR-34a and thus upregulates ULBP2 expression in liver CSCs, leading to CSC killing by NK cells (Weng J. et al., 2019). This suggests that the c-Myc/CD44-mediated ceRNA regulatory network may be more complex than previously thought. In addition, PD-L1 and LDHA have been reported to act as ceRNAs to promote the expression and functions of each other through competing for the binding of miR-34a in TNBC (Huang et al., 2017). These studies on ceRNAs suggest that single therapeutic modality, whether immunotherapy or miR-34a alone, may not fully target both bulk cancer cells and CSCs, whereas combination of miR-34a with immunotherapy or other debuking therapeutic regimens will likely elicit synergetic therapeutic effects for cancer treatment.

### LncRNAs as miR34a ceRNAs

Long non-coding RNAs, typically hundreds to thousands of nucleotides in length, play important regulatory functions in gene expressions and molecular processes. Recent studies have implicated several lncRNAs in regulating stemness, EMT, and metastasis via acting as miR-34a-sponging ceRNAs. For instance, cancer-associated MSCs contribute to HCC stemness and tumorigenesis, mediated in part by lncRNA MUF acting as a ceRNA for miR-34a and subsequently promoting tumor sphere formation and EMT as a result of the upregulated miR-34a target Snail1 (Yan et al., 2017). Likewise, LINC00346 functions as a sponge for miR-34a and promotes cancer cell proliferation, invasion, and metastasis in gastric cancer by protecting NOTCH1, AXL, and CD44 from miR-34a-mediated degradation (Xu et al., 2019). In addition, the oncogenic TFs KLF5 and MYC could bind to the *LINC00346* promoter and enhance its expression (Xu et al., 2019). The lncRNA XIST serves as a miR-34a ceRNA via competing with MET for miR-34a binding and thus promoting cancer cell proliferation and tumor growth through upregulated MET–PI3K–AKT signaling (Liu et al., 2018). Consistently, MET expression was positively correlated with XIST but negatively with miR-34a levels in tumor specimens (Liu et al., 2018). As yet another example, lncRNA NEAT1 promoted CRC cell proliferation and metastasis by serving as a miR-34a ceRNA, leading to subsequent repression of the miR-34a/SIRT1 axis and activation of the Wnt/β-catenin signaling (Luo et al., 2019).

### Pseudogenes as miR-34a ceRNAs

Pseudogenes, the so-called “junk” genes, do not encode functional proteins, but their transcription can be either activated or silenced in cancer (Pink et al., 2011). A recent study showed that ASS1P3, a pseudogene of argininosuccinate synthase 1 (ASS1) gene, could function as a ceRNA to regulate its cognate gene and suppress cancer cell proliferation by competing with miR-34a (Wang et al., 2019). In this scenario, miR-34a, intriguingly, appeared to function as an oncogene.

### Circular RNAs as miR-34a ceRNAs

Circular RNAs (circRNAs) are closed RNA transcripts with the 5′-terminus covalently linked to the 3′-terminus, resulting in a scrambled exon order. Previously, circRNAs were considered as errors of splicing. However, emerging evidence suggests that circRNAs may have important roles in regulating cellular functions. For example, circBIRC6, enriched in the AGO2 complex, can directly interact with miR-34a as a sponge to maintain human embryonic stem cell pluripotency and suppress miR-34a-mediated differentiation (Yu et al., 2017). Similarly, circINSR, by acting as a sponge of miR-34a, can promote proliferation and reduce apoptosis of embryonic myoblasts through derepression of miR-34a target genes such as Bcl-2 and cyclin E2 (Shen et al., 2020). These studies, collectively, suggest that circRNAs may function as regulators of embryonic development in which miR-34a plays a vital role. In cancer as well, circRNAs can upregulate target genes of miR-34a and promote cancer progression by acting as a miR-34a sponge (He et al., 2017; Chen Y. et al., 2019). CircASH2L promoted tumor invasion, proliferation, and angiogenesis by sponging out miR-34a and upregulating Notch-1 (Chen Y. et al., 2019). In patient specimens, circASH2L expression positively correlated with lymphatic invasion and tumor–node–metastasis (TNM) stage (Chen Y. et al., 2019), suggesting the clinical relevance for predicting prognosis.

## miR-34a-Based Therapeutic Strategies in Cancer Treatment

### Lessons Learned From the Clinical Trial of MRX34

The first-in-class phase I clinical trial of MRX34, a liposomal miR-34a mimic, was initiated in 2013 (NCT01829971), which, unfortunately, was halted by Food and Drug Administration (FDA) in September of 2016 due to severe immune-mediated toxicities and four patient deaths in the expansion cohort. Still, this first clinical trial of miRNA-based cancer therapy provided valuable insights for future development of this class of drugs.

One consideration is the packaging vehicle. The delivery system used in this clinical trial is SMARTICLE, a liposomal nanoparticle with a diameter of ∼110 nm (Beg et al., 2017). Although this liposomal nanoparticle design fulfills the high efficiency of miR-34a uptake and long circulation time in blood, it does not directly target the delivery of miR-34a specifically to cancer cells. It has been established that MRX34, when administered intravenously to mice and non-human primates, delivers a high amount of miR-34a mimic to the liver and many other tissues and organs including bone marrow, spleen, and lung (Beg et al., 2017). This non-specific target delivery could be one of the potential reasons behind the prematurely terminated trial.

Another consideration is the drug dose. In the initial stage of this clinical trial, the dose was started at 10 mg/m^2^ and then escalated to 20, 33, 50, 70, 93, and 124 mg/m^2^ (Beg et al., 2017). However, the 124 mg/m^2^ dose was determined to be above the maximum tolerated dose (MTD) due to systemic inflammatory response syndrome observed in patients. Subsequently, the dose was amended to 110 mg/m^2^, and the drug was administered along with dexamethasone premedication in week 1 aiming to dampen immune response (Hong et al., 2020). However, this amended schedule was still associated with serious adverse effects (AEs) and no apparent increase in efficacy. It suggests that the safety window of miR-34a seems small, and administration of high dose of miR-34a may not be clinically beneficial due to treatment-induced immune responses. Thus, the dose and dosing schedule of miR-34a in patients must be carefully evaluated and controlled.

More importantly, the exact nature of immune-related AEs in the miR-34a trial patients should be elucidated. In the latest report on MRX34a clinical trial, the serious AEs included sepsis, hypoxia, cytokine release syndrome, and hepatic failure, a pattern suggestive of immune-mediated toxicity (Hong et al., 2020), which could include liposomal-related, double-stranded RNA (dsRNA)-related non-specific inflammation, and miR-34a-related specific modulation of gene expression. Unfortunately, the preclinical AE profile performed with MRX34 in animals, including non-human primates, did not predict the immune activation profile in humans (Hong et al., 2020).

### Delivery Systems of miR-34a

**FIGURE 5:**
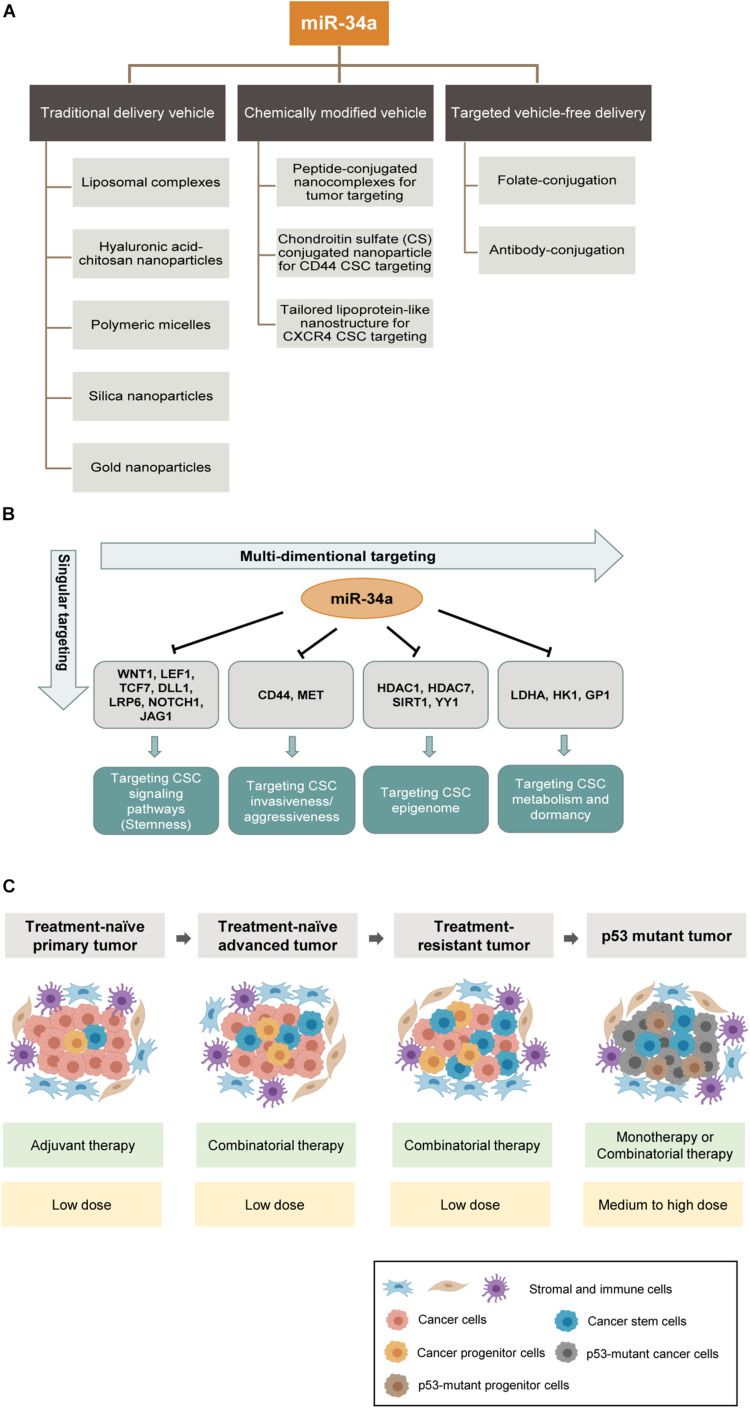
MicroRNA-34a-based therapeutic strategies. **(A)** Shown are three main categories of preclinical miR-34a delivery platforms: conventional delivery vehicles, chemically modified vehicles and targeted vehicle-free delivery. Some examples are shown below. **(B)** Current anti-CSC therapeutic strategies primarily target a singular aspect of CSCs. In contrast, miR-34a based therapy can potentially offer multidimensional antitumor effects because of its wide range of targets. **(C)** Potential clinical settings in which miR-34a mimics can be deployed as an anticancer therapeutic. The miR-34a based therapeutic strategies are shown in green boxes and dosing recommendations of miR-34a are indicated in yellow boxes below.

An ideal delivery system for miR-34a replacement therapy should achieve three main goals: (1) efficient delivery with high cellular uptake and stability; (2) specific delivery to tumors while avoiding or minimizing non-specific delivery to other normal tissues; and (3) minimizing systemic immune responses and immunotoxicities. In the past decade, miR-34a has been successfully delivered in mouse models using various methods, including viral vectors and non-viral vectors. Nevertheless, most studies have focused on non-viral approaches due to the high immunogenicity and the risk of insertional mutagenesis from viral vectors. The non-viral vectors reported for miR-34a delivery include, among others, liposomal complexes, hyaluronic acid–chitosan nanoparticles, polymeric micelles, silica nanoparticles, and gold nanoparticles (Trang et al., 2011; Tivnan et al., 2012; Deng et al., 2014; Salzano et al., 2016; Cai et al., 2020; Figure 5A).

One of the major challenges in miRNA delivery is the toxicities associated with the packaging vehicles due to non-specific biodistribution. To overcome this barrier, one approach is to chemically modify the vehicle for tumor-targeted delivery. For example, Hu and colleagues designed peptide-conjugated nanocomplexes by chemically conjugating CC9, a specific tumor-homing and tumor-penetrating bifunctional peptide, to the cationic β-cyclodextrin-polyethylenimine vectors for efficiently delivering miR-34a to tumors (Hu et al., 2013). CC9 homes in on tumor cells through the arginine–glycine–aspartic acid (RGD) motif and is then proteolytically cleaved in the tumor to produce CRGDK, which has high affinity for neuropilin-1 (NRP-1), and NRP-1 binding subsequently triggers tissue penetration (Hu et al., 2013). Another strategy is to chemically conjugate poly(amidoamine) with chondroitin sulfate (CS) to promote CD44-mediated endocytosis. CS is a natural polysaccharide with high affinity for CD44, which is frequently overexpressed in solid tumors and CSCs; consequently, CS conjugated to nanoparticles can act as a specific ligand to target metastatic cancer cells and CSCs via CD44-mediated endocytosis (Liang et al., 2015). Yet another example of CSC-specific targeting is to exploit the chemokine receptor CXCR4, which is highly expressed on glioma-initiating cells (GICs) compared to differentiated glioma cells. Stromal-cell-derived factor 1 (SDF1 or CXCL12) could bind to CXCR4 and thus stimulate the macropinocytosis pathway, which works as a mechanism for targeted delivery. A novel stimulated lipoprotein-like nanoparticle platform was designed to incorporate an α-helix sequence, which assembles SDF1-mimicking peptide and exhibits strong binding to CXCR4 (Jiang et al., 2020). Therein, by enhancing micropinocytosis induced by CXCR4 activation, this nanoparticle achieves efficient accumulation in GICs and suppresses glioma stemness and drug resistance *in vitro* and *in vivo* (Jiang et al., 2020; Figure 5A).

Although these chemically modified vehicles, to some extent, improve the efficiency and specificity of delivery, vehicle-related problems such as systemic toxicity and clearance likely still limit the full manifestation of the therapeutic potential of miRNA drugs. In theory, using a targeted, vehicle-free delivery approach may eliminate the vehicle-associated problems once and for all (Figure 5A). One prominent approach is ligand-conjugated miRNA delivery. In a recent study, the Kasinski group developed a folate-conjugated miR-34a. They directly conjugated synthetic miR-34a mimics to folate (FolamiR), the ligand of the folate receptor that is overexpressed in many cancer cells, to deliver functionally active miR-34a (Orellana et al., 2017). With this folate-conjugated delivery system, miR-34a is selectively targeted to the tumor area, enters tumor cells through binding to the high-affinity folate receptor, downregulates target genes, and suppresses the growth *in vivo* of mouse models of human lung and breast cancers (Orellana et al., 2017).

Even package-free delivery of ligand-conjugated miRNAs still faces one of the challenges in the delivery field, endosomal sequestration. To tackle this challenge, the Kasinski team developed a novel method to promote endosomal escape of delivered miRNA (Orellana et al., 2019). Relying on the difference in solute contents between nascent endosomes and the cytoplasm, they utilized a small molecule, nigericin, that exchanges K^+^ for osmotically inactive proton (H^+^) to achieve balance in charge without release of a compensatory solute (Na^+^), thus causing an osmotic differential that leads to endosome swelling and bursting. They found that folate–nigericin–miR-34a caused increased endosomal release of miR-34a and promoted miR-34a targeting and engagement in RNA-induced silencing complex (RISC) (Orellana et al., 2019). This endosomal escape strategy could provide higher efficacy and further reduce toxicity while decrease the dosage of miR-34a mimics needed to achieve effective gene modulation.

### miR-34a-Based Therapeutic Strategies

A major clinical challenge in cancer treatment is therapy resistance, and CSCs have been established as a major reservoir of cancer cells that mediate resistance. For example, CSCs protect themselves from ionizing radiation and certain chemotherapy drugs by reducing ROS levels induced by clinical treatments (Diehn et al., 2009). Therefore, there has been an intensive effort to develop therapeutic strategies to effectively target CSCs. Unfortunately, many potential anti-CSC drugs have not fared well in clinical trials (Lytle et al., 2018), and several reasons may have contributed to such failures (Clarke, 2019). First of all, some drugs were developed based on activity against cells that were incorrectly identified as CSCs. Second, heterogeneity exists within the CSC population, and cycling and quiescent CSCs differ in cell surface markers and signaling pathways. Third, CSC plasticity makes it even more challenging for CSC-targeting therapies to work, as some non-CSCs and tumor progenitor cells can undergo reprogramming and dedifferentiate to CSCs. Fourth, some “CSC-targeting” therapeutics do not specifically aim at just CSCs but have significant activities against normal SCs, thus eliciting unwanted toxicities. Lastly, CSCs exploit multiple signaling pathways for the maintenance of stemness and survival, but current anti-CSC therapeutics may only target one specific pathway, i.e., singular dimension (Figure 5B). On the contrary, miR-34a would be a “multi-dimensional” anti-CSC therapeutic that can simultaneously target several critical CSC signaling pathways (Figure 5B). Bearing in mind of the multiple innate survival mechanisms of CSCs, it is evident that successful elimination of CSCs will depend on combination strategies that, ideally, target both bulk non-CSCs and heterogeneous and dynamically evolving CSC subpopulations, as well as the core stemness program (i.e., signaling pathways) in CSCs.

From the first-in-human miR-34a clinical trial, we have learned that miR-34a dosage and administration are tightly constrained by immune-related toxicities. Thus, combining miR-34a with other therapeutic modalities should maximize treatment benefits while minimizing miR-34a dosage-associated immunotoxic AEs. Under this principle, we provide four clinical settings where miR-34a can potentially be deployed to therapeutically target CSCs aiming to delay therapy resistance, overcome resistance, and prevent or inhibit tumor relapse (Figure 5C).

In the first scenario, in treatment-naive primary tumors, especially the early-stage tumors, CSCs generally represent a small subpopulation within the tumor bulk, and most of them are quiescent, whereas non-CSCs are the main population. Accordingly, the standard-of-care therapy, such as androgen-deprivation therapy and radiation (for prostate cancer), should be considered as the main approach to eliminate non-CSCs, while low-doses of miR-34a therapy can be deployed as an adjuvant to tackle residual therapy-resistant CSCs and to prevent relapse (Figure 5C). In principle, low doses of miR-34a might also be utilized simultaneously with the standard of care therapy, to tackle the preexisting cancer cell heterogeneity and thus inhibit therapy resistance and relapse.

Another clinical setting is untreated advanced tumors, i.e., high-grade and metastatic tumors. In this setting, the CSC composition would be significantly increased compared to primary low-grade tumors, and CSCs have become a clinically relevant subpopulation. Hence, conventional therapies that only target non-CSCs would not suffice. At this stage, perceived clinical use of miR-34a would be via a combinatorial approach, in conjunction with the standard of care therapies (Figure 5C).

The third clinical setting is treatment-failed and therapy-resistant tumors, in which the CSC population would further increase as therapies may reprogram some differentiated cancer cells to stem-/progenitor-cell-like state. In this setting, miR-34a replacement therapy would be utilized in combination with chemotherapy and/or targeted therapy (Figure 5C). The miR-34a-based combinatorial therapy would overcome therapy resistance and drive a more efficient therapeutic effect by cotargeting both CSCs and bulk tumor cells. The proof of concept has been shown in some recent studies. For example, the combination therapy of docetaxel and rubone, a miR-34 activator, can reverse chemoresistance, suppress the CSC population, and enhance tumor regression in taxane-resistant PCa (Lin et al., 2019). As for targeted therapy resistance, the combination of miR-34a and let-7 with erlotinib, a TKI for NSCLC, improves the therapeutic efficacy *in vivo*, which suggests combination of several antitumor miRNAs as another angle of combinational strategy (Stahlhut and Slack, 2015).

The final clinical setting is the application of miR-34a in p53-mutant tumors (Figure 5C). p53 mutation occurs in > 50% of human malignancies (Perri et al., 2016) and often correlates with tumor aggressiveness and poor patient survival (Robles and Harris, 2010). In p53 mutant tumors, most cancer cells possess some CSC properties (Figure 5C). Considering that miR-34a is a direct transcriptional target of p53 and a major mediator of all p53-driven antitumor effects including apoptosis, senescence, and differentiation, miR-34a could potentially become the primary therapeutic agent for p53 mutant tumors (Figure 5C). In those tumors without functional p53, miR-34a replacement would elicit potent antitumor efficacy via bypassing p53. In such tumors, miR-34a could be employed as either monotherapy or combinatorial therapy together with chemo- or targeted therapeutics (Figure 5C).

## Concluding Remarks

MicroRNA-34a, a potent CSC inhibitor, has been demonstrated to be a potential anticancer therapeutic for the treatment of various cancers. The lessons, derived from the first-in-human clinical trial of miR-34a, suggest that we need to better understand the effects of miR-34a on the cells in the TME, especially various immune cells and tumor immune esponses. Apparently, the delivery strategy for optimal miR-34a delivery to the tumor will continue to be a challenge for its clinical translation. Although we have made some progress on better uptake and specific tumor targeting through novel ligand-conjugation approaches, the endosomal sequestration and toxicity remain obstacles to overcome. Despite these challenges, we envision novel miR-34a-based combinatorial protocols to be developed in the near future to tackle cancer cell heterogeneity and plasticity and inhibit therapy resistance.

## Author Contributions

WL collected data from published studies and wrote the manuscript. YW, RL, AK, HS, FS, and DT discussed the review content and critically reviewed the manuscript draft. DT aided in manuscript writing and finalized the manuscript. All authors have read and agreed to the published version of the manuscript.

## Conflict of Interest

The authors declare that the research was conducted in the absence of any commercial or financial relationships that could be construed as a potential conflict of interest.

## Abbreviations

AE: adverse effect
ALDH: aldehyde dehydrogenase
AML: acute myeloid leukemia
ARG1: arginase 1
ASS1: Argininosuccinate synthase 1
BCSC: breast cancer stem cell
C/EBPα: CCAAT enhancer binding protein alpha
CAF: cancer-associated fibroblast
CCSC: colon cancer stem cell
ceRNA: competing endogenous RNA
CLL: chronic lymphocytic leukemia
CML: chronic myeloid leukemia
CS: chondroitin sulfate
CSC: cancer stem cell
CTL: cytotoxic T lymphocyte
DGKζ: diacylglycerol kinase ζ
DLL1: delta-like 1
EMT: epithelial-to-mesenchymal transition
ESA: epithelial specific antigen
EV: extracellular vesicle
EZH2: enhancer of zeste homolog 2
FolamiR: folate-conjugated miRNA
GSC: glioma stem cell
HCC: hepatocellular carcinoma
HNSCC: head and neck squamous cell carcinoma
HSC: hematopoietic stem cell
IL-6R: IL-6 receptor
iNOS: inducible nitric oxide synthase
LDHA: lactate dehydrogenase A
LncRNA: long non-coding RNA
MB: medulloblastoma
MDSC: myeloid-derived suppressor cell
miRNA: microRNA
MK: megakaryocyte
MM: multiple myeloma
MRE: microRNA recognition element
MSC: mesenchymal stem cells
MTD: maximum tolerated dose
NK cell: natural killer cell
NRP-1: Neuropilin-1
NSCLC: non-small cell lung cancer
OncomiR: oncogenic miRNA
PCa: prostate cancer
PCSC: prostate cancer stem cell
PRC2: polycomb repressive complex 2
PRKD1: protein kinase D1
SC: stem cell
SCF: stem cell factor
SCLC: small cell lung cancer
SDF1: stromal cell-derived factor 1
SP: Side Population
TA cell: transit-amplifying cell
TAM: tumor-associated macrophage
TFs: transcription factors
TGIF2: transforming growth interaction factor 2
Th17 cell: T helper 17 cell
TIL: tumor-infiltrated lymphocyte
TiME: tumor immune microenvironment
TKI: tyrosine kinase inhibitor
TME: tumor microenvironment
TNBC: triple negative breast cancer
TNF-α: tumor necrosis factor-α
TPC: tumor propagating cells
Treg: regulatory T cell
UTR: untranslated region
YY1: Yin Yang-1.
