# Supplementary material for: MicroRNA-34a: Potent Tumor Suppressor, Cancer Stem Cell Inhibitor, and Potential Anticancer Therapeutic
Source: Front Cell Dev Biol. 2021 Mar 8;9:640587. doi: 10.3389/fcell.2021.640587 (PMC7982597; doi:10.3389/fcell.2021.640587)
Supplement: Supplementary file 1 [file Table_1.docx]

# Supplementary Table 1. Reported miR-34a targets to date (as of Nov. 2020).

| **Validated target gene** | **Full name** | **Cellular process** | **Biological effect associated with miR-34a-induced gene silencing** | **References** |
| --- | --- | --- | --- | --- |
| ACSL1 | Acyl-CoA Synthetase Long-Chain Family Member 1 | Metabolism | Regulates lipid metabolism | Li et al. 2011 |
| ADAM10 | Adam Metallopeptidase Domain 10 | Immunity | Modulates T cell regulatory networks | Hart et al. 2019 |
| ADAM12 | Adam Metallopeptidase Domain 12 | Metastasis | Inhibits metastasis | Huang et al. 2018 |
| ADIPOR2 | Adiponectin Receptor | Metabolism | Regulates lipid metabolism | Wen et al. 2018 |
| ALDH2 | Aldehyde Dehydrogenase 2 | Cell survival | Anti-apoptotic | Fan et al. 2013 |
| ANG1 | Angiopoietin-1 | Cell survival | Enhance apoptosis | Syed et al. 2017 |
| ANK3 | Ankyrin-3 | Differentiation | Regulates synaptic function; impairs neuronal differentiation | Bavamian et al. 2015 |
| AR | Androgen Receptor | Differentiation | Represses cancer cell proliferation | O¨ stling et al. 2011; Kashat et al. 2012 |
| ARHGAP1 | Rho GTPase Activating Protein 1 | Invasion | Inhibits cell migration and invasion | Ahn et al. 2012 |
| ATG4B | Autophagy Related 4b Cysteine Peptidase | Cell survival | Inhibits autophagy | Rothe et al. 2014 |
| ATG5 | Autophagy-Related Gene 5 | Cell survival | Inhibits cell proliferation, apoptosis, migration, invasion, and autophagy | Cheng et al. 2019 |
| ATG9A | Autophagy-Related 9a | Cell survival | Inhibits autophagy | Huang et al. 2014; Morgado et al. 2015 |
| AXIN2 | Axin 2 | Stemness | Inhibits Wnt signaling | Kim et al. 2013 |
| AXL | AXL Receptor Tyrosine Kinase | Invasion | Inhibits cell migration and invasion | Kaller et al. 2011; Mackiewicz et al. 2011; Mudduluru et al. 2011 |
| BAX | Bcl-2-Associated X | Cell survival | Anti-apoptosis | Fan et al. 2016 |
| BCL2 | B-Cell Leukemia/Lymphoma 2 | Cell survival | Induces apoptosis | Bommer et al. 2007; He et al. 2007; Cole et al. 2008 |
| BCL-6 | B-Cell Leukemia/Lymphoma 6 | Immunity | Anti-inflammatory | Bernardo et al. 2012 |
| BIRC5 | Survivin | Cell survival | Induces apoptosis | Kaller et al. 2011; Shen et al. 2012 |
| CACNB3 | Calcium Channel Subunit Beta-3 | Differentiation | Regulates synaptic function; impairs neuronal differentiation | Bavamian et al. 2015 |
| CCL22 | C–C Motif Chemokine 22 | Immunity | Inhibits tumor growth | Yang et al. 2012 |
| CCND1 | Cyclin D1 | Cell cycle | G1-arrest | Fujita et al. 2008; Sun et al. 2008 |
| CCNE1 | Cyclin E1 | Cell cycle | G1-arrest | Han et al. 2015 |
| CCNE2 | Cyclin E2 | Cell cycle | G1-arrest | He et al. 2007; Toyota et al. 2008 |
| CCR1 | C-C Chemokine Receptor Type 1 | Immunity | Modulates T cell regulatory networks | Hart et al. 2019 |
| c-KIT | V-Kit Hardy-Zuckerman 4 Feline Sarcoma | Stemness | Inhibits cell proliferation and survival | Siemens et al. 2013; Pu et al. 2016 |
| CD11A | Integrin Subunit Alpha L | Immunity | Modulates T cell regulatory networks | Hart et al. 2019 |
| CD24 | Cluster Of Differentiation 24 | Metastasis | Inhibits metastasis | Muppala et al. 2013 |
| CD44 | Heparan Sulfate Proteoglycan | Stemness | Inhibits CSC and metastasis | Liu et al. 2011 |
| CD47 | Cluster Of Differentiation 47 | Immunity | Enhance phagocytosis | Junker et al. 2009 |
| CDK4 | Cyclin-Dependent Kinase 4 | Cell cycle | G1-arrest | He et al. 2007; Toyota et al. 2008 |
| CDK6 | Cyclin-Dependent Kinase 6 | Cell cycle | G1-arrest | He et al. 2007; Toyota et al. 2008 |
| CRTC1 | CREB Regulated Transcription Coactivator 1 | Metabolism | Regulates lipid metabolism | Kim et al. 2016 |
| CSF1R | Colony Stimulating Factor 1 Receptor | Metastasis | Inhibits metastasis | Shi et al. 2020 |
| CTNNB1 | Beta-Catenin | Stemness | Inhibits Wnt signaling | Kim et al. 2011 |
| DCX | Doublecortin | Differentiation | Regulates synaptic function; impairs neuronal differentiation and neuroblast migration | Mollinari et al. 2015 |
| DGKΖ | Diacylglycerol Kinase Ζ | Immunity | DAG-mediated signaling, T cell activation | Shin et al. 2013 |
| DLL1 | Notch Ligand Delta-Like 1 | Stemness | Inhibits cell proliferation | de Antonellis et al. 2011 |
| E2F3 | E2F Transcription Factor 3 | Cell cycle | Inhibits cell proliferation | Tazawa et al. 2007; Welch et al. 2007 |
| EGFR | Epidermal Growth Factor Receptor | Differentiation | Inhibits cancer cell proliferation | Li et al. 2017b |
| ERBB2 | V-Erb-B2 Avian Erythroblastic Leukemia Viral Oncogene Homolog 2 | Differentiation | Suppresses cell proliferation and invasion | Wang et al. 2017 |
| FGFR1 | Fibroblast Growth Factor Receptor 1 | Metabolism | Regulates lipid metabolism | Fu et al. 2014 |
| FLOT2 | Flotillin 2 | Invasion | Inhibits cell migration and invasion | Liu et al. 2015 |
| FNDC3B | Fibronectin Type III Domain Containing 3B | Invasion | Inhibits cell migration and invasion | Yang et al. 2017 |
| FNDC5 | Fibronectin Type III Domain-Containing Protein 5 | Metabolism | Promotes diet-induced obesity | Ge et al. 2017 |
| FOXJ2 | Forkhead Box J2 | Differentiation | Promotes endothelial differentiation | Chen et al. 2014 |
| FOXP1 | Forkhead Box P1 | Differentiation | Blocks in B cell development | Rao et al. 2010 |
| FOXP2 | Forkhead Box Protein P2 | Differentiation | Regulates axonal growth | Jia et al. 2018 |
| FOXP3 | Forkhead Box P3 | Metastasis | Inhibits metastasis | Xi et al. 2018 |
| FRA-1 | Fos-Related Antigen 1 | Invasion | Inhibits cell migration and invasion | Wu et al. 2012; Yang et al. 2013 |
| FRAT1 | FRAT Regulator Of WNT Signaling Pathway 1 | Cell survival | Inhibits cell proliferation and promotes apoptosis | Werner et al. 2017 |
| FUT8 | Fucosyltransferase 8 | Metabolism | Decreases fucosylation and represses hepatocarcinogenesis | Bernardi et al. 2013 |
| GAS1 | Growth Arrest Specific1 | Cell survival | Cell proliferation and inhibition of apoptosis | Ma et al. 2013 |
| GATA2 | GATA-Binding Factor 2 | Differentiation | Restricts the acquisition of expanded cell fate potential in pluripotent stem cells | Choi et al. 2017 |
| GOLPH3 | Golgi Phosphoprotein 3 | Stemness | Inhibits stemness and drug resistance | Zhang et al. 2017 |
| GP1 | Glucose-6-Phosphate Isomerase | Metabolism | Controls glucose metabolism | Kim et al. 2013 |
| HDAC1 | Histone Deacetylase 1 | Epigenetics | Inhibits tumor progression, cell proliferation, and cell cycle progression | Zhao et al. 2013; Wu et al. 2014 |
| HDAC7 | Histone Deacetylase 7 | Epigenetics | Inhibits tumor progression, cell proliferation, and cell cycle progression | Wu et al. 2014 |
| HK1 | Hexokinase 1 | Metabolism | Controls glucose metabolism | Kim et al. 2013 |
| HK2 | Hexokinase 2 | Metabolism | Controls glucose metabolism | Kim et al. 2013 |
| HMGA2 | High Mobility Group AT-Hook 2 | Epigenetics | Inhibits cell growth and differentiation, and induces senescence | Tazawa et al. 2007; Welch et al. 2007 |
| HMGB1 | High Mobility Group Box 1 | Epigenetics | Inhibits cell invasion, and induces apoptosis | Liu et al. 2014; Chandrasekaran et al. 2016 |
| HNF-4G | Hepatocyte Nuclear Factor 4 Gamma | Invasion | Inhibits cell migration and invasion | Sun et al. 2015 |
| HNF-4Α | Hepatocyte Nuclear Factor-4a | Metabolism | Regulates lipid and lipoprotein metabolism | Wang and Burke 2013; Xu et al. 2015 |
| HOTAIR | HOX Transcript Antisense RNA | Epigenetics | Inhibits tumor progression and EMT | Chiyomaru et al. 2013; Liu et al. 2015 |
| HSP70 | Heat Shock Protein 70 | Stemness | Inhibits SC survival | Feng et al. 2014 |
| IL-6R | Interleukin 6 Receptor | EMT | Inhibits EMT | Rokavec et al. 2014 |
| IMPDH | IMP (Inosine5-Monophosphate) Dehydrogenase | Metabolism | Downregulates purine metabolism | Kim et al. 2012 |
| KLF4 | Kruppel-Like Factor 4 | Invasion | Inhibits cell migration and invasion | Lopez et al. 2018 |
| L1CAM | L1 Cell Adhesion Molecule | Invasion | Inhibits cell migration and invasion | Schirmer et al. 2014 |
| LDHA | Lactate Dehydrogenase A | Metabolism | Downregulates glucose metabolism | Kaller et al. 2011; Xiao et al. 2016 |
| LEF1 | Lymphoid Enhancer Binding Factor 1 | Stemness | Inhibits Wnt signaling | Kaller et al. 2011; Kim et al. 2011 |
| LGR4 | Leucine Rich Repeat Containing G Protein-Coupled Receptor 4 | Metastasis | Inhibits metastasis | Hou et al. 2016; Wu et al. 2020 |
| LIN28A | Lin-28 Homolog A | Stemness | Prevents backsliding to pluripotency and promotes differentiation | Jain et al. 2012 |
| LRP6 | Low-Density Lipoprotein Receptor-Related | Stemness | Inhibits Wnt signaling | Kim et al. 2011 |
| LYDGI | RhoGDI2, D4-GDI, RhoGDIβ | Cell survival | Enhances apoptosis | Duan et al. 2015 |
| MAGE-A | Mage-A Tumor-Associated Antigens | Cell survival | Apoptosis; positive feedback of p53 | Weeraratne et al. 2011 |
| MAPT | Microtubule-Associated Protein Tau | Differentiation | Downregulates tau expression | Dickson et al. 2013 |
| MAT2A | Methionine Adenosyltransferase 2A | Invasion | Inhibits cell migration and invasion | Tomasi et al. 2017 |
| MDM4 | MDM4 Regulator of P53 | Cell cycle | Inhibits cell proliferation | Mandke et al. 2012 |
| MET | Met Proto-Oncogene | Invasion | Inhibits cell migration and invasion | He et al. 2007; Li et al. 2009 |
| MIEF1 | Mitochondrial Elongation Factor 1 | Cell cycle | Inhibits cell proliferation | Chen et al. 2018 |
| MIEF2 | Mitochondrial Elongation Factor 2 | Cell cycle | Inhibits cell proliferation | Chen et al. 2018 |
| MMP14 | Matrix Metalloproteinase-14 | Invasion | Inhibits cell migration and invasion | Jia et al. 2014 |
| MMP2 | Matrix Metalloproteinase-2 | Invasion | Inhibits cell migration and invasion | Yang et al. 2017 |
| MMP9 | Matrix Metalloproteinase-9 | Invasion | Inhibits cell migration and invasion | Jia et al. 2014 |
| MMSET | Multiple Myelomas Set Domain | Metastasis | Inhibits metastasis | Dong et al. 2018 |
| MSI1 | RNA-Binding Protein Musashi Homolog 1 | Stemness | Inhibits cell proliferation | Vo et al. 2011 |
| MTA2 | Metastasis Associated 1 Family Member 2 | Invasion | Inhibits cell migration and invasion | Kaller et al. 2011 |
| MYB | V-Myb Myeloblastosis Viral Oncogene | Differentiation | Promotes differentiation | Navarro et al. 2009 |
| MYC | V-Myc Myelocytomatosis Viral Oncogene | Stemness | G1-arrest | Cole et al. 2008; Wei et al. 2008 |
| MYCN | V-Myc Myelocytomatosis Viral Related | Cell cycle | G1-arrest | Cole et al. 2008 and Wei et al. 2008 |
| NAMPT | Nicotinamide Phosphoribosyltransferase | Metabolism | Downregulates NAD^+^ biosynthesis | Choi et al. 2013 |
| NLRC5 | Nlr Family Card Domain Containing 5 | Immunity | Positively regulates NF-κB signaling by promoting the nuclear translocation of p65 | Li et al. 2016; Periyasamy et al. 2019 |
| NOTCH1 | Notch Homolog 1 | Stemness | Inhibits Notch signaling | Li et al. 2009; Pang et al. 2010 |
| NOTCH2 | Notch Homolog 2 | Stemness | Inhibits Notch signaling | Kwon et al. 2017 |
| NUMB | Numb | Stemness | Inhibits stemness | Bu et al. 2016 |
| OCT4 | Octamer-Binding Transcription Factor 4 | Stemness | Inhibits stemness | Ng et al. 2014; Zhang et al. 2016 |
| PAI-1 | Plasminogen Activator Inhibitor 1 | Metastasis | Inhibits metastasis | Lin et al. 2017; Öner et al. 2018 |
| PDGFRΑ | Platelet-Derived Growth Factor Receptor, Alpha | Invasion | Inhibits cell migration and invasion | Garofalo et al. 2013 |
| PDGFRΒ | Platelet-Derived Growth Factor Receptor, Beta | Invasion | Inhibits cell migration and invasion | Garofalo et al. 2013 |
| PDK1 | Pyruvate Dehydrogenase Kinase 1 | Metabolism | Downregulates glucose metabolism | Kim et al. 2013 |
| PD-L1 | Programmed Death-Ligand 1 | Immunity | Downregulates PD-L1 expression and promotes T cell responses | Wang et al. 2015; Cortez et al. 2015 |
| PEBP4 | Phosphatidylethanolamine-Binding Protein 4 | EMT | Inhibits EMT | Yu et al. 2014 |
| POFUT1 | Protein O-Fucosyltranferase 1 | Metabolism | Inhibits cell survival and cardiac repair | Bernardo et al. 2012 |
| PPP1CC | Protein Phosphatase 1γ | Cell survival | Regulates DNA repair and enhance ATM signaling and cell death | Takeda et al. 2015 |
| PPARΑ | Peroxisome Proliferator-Activated Receptor-Α | Metabolism | Promotes liver steatosis | Ding et al. 2015 |
| PPP1R11 | Protein Phosphatase 1 Regulatory Subunit 11 | EMT | Inhibits EMT | Li et al. 2017a |
| PRF1 | Perforin 1 | Immunity | Modulates T cell regulatory networks | Hart et al. 2019 |
| PRKD1 | Protein Kinase D1 | Stemness | Inhibits Wnt signaling | Kim et al. 2016 |
| RAD51 | RAD51 recombinase | Cell survival | Inhibits DSB repair and promotes radiosensitivity | Cortez et al. 2015 |
| RBP2 | Retinoblastoma Binding Protein 2 | Differentiation | Promotes osteogenic Differentiation | Fan et al. 2016 |
| RCAN1 | Regulator of Calcineurin 1 | Immunity | Modulate endothelial inflammation | Yuan et al. 2017 |
| RICTOR | Rapamycin-Insensitive Companion of Mammalian Target of Rapamycin | Metabolism | Suppresses cell proliferation and tumor growth | Rathod et al. 2014 |
| RRAS | Related RAS Viral Oncogene Homolog | Invasion | Inhibits cell migration and invasion | Kaller et al. 2011 |
| RXRΑ | Retinoid X Receptor Α | Metabolism | Promotes liver fibrosis | Oda et al. 2014 |
| SATB1 | Special AT-Rich Sequence-Binding Protein-1 | Invasion | Inhibits cell migration and invasion | Liu et al. 2019 |
| SEMA4B | Semaphorin 4B | Differentiation | Anti-inflammatory | Bernardo et al. 2012 |
| S-IL-6R | Soluble Interleukin 6 Receptor | Immunity | Inhibits IL-6 trans-signaling and downregulates inflammation | Li et al. 2015 |
| SIRT1 | Sirtuin 1, Silent Information Regulator 1 | Epigenetics | Increases p53 acetylation and activation and induces apoptosis | Yamakuchi et al. 2008; Kojima et al. 2010 |
| SIRT6 | Sirtuin 6 | Epigenetics | Promotes differentiation | Lefort et al. 2013; Dotto et al. 2014 |
| SMAD4 | Mothers Against Decapentaplegic Homolog 4 | EMT | Inhibits EMT | Qiao et al. 2015 |
| SNAIL | Snail Family Zinc Finger 1 | EMT | Inhibits EMT | Siemens et al. 2011 |
| SOX7 | SRY-Box Transcription Factor 7 | Cell cycle | Inhibits cell proliferation and induces apoptosis | Yang et al. 2018 |
| SRC | SRC proto-oncogene, non-receptor tyrosine kinase | Cell survival | Inhibits cell cycle progression and migration, and induces apoptosis | Muppala et al. 2013 |
| STIM1 | Stromal Interaction Molecule 1 | Immunity | Regulates T-cell activation signaling | Diener et al. 2018 |
| STX1A | Syntaxin-1A | Differentiation | Regulates synaptic function; impairs neuronal differentiation | Agostini et al. 2011 |
| SYT1 | Synaptotagmin-1 | Differentiation | Regulates synaptic function; impairs neuronal differentiation | Agostini et al. 2011 |
| TCF1 | T-Cell Factor 1 | Metastasis | Inhibits metastasis | Liu et al. 2018 |
| TCF7 | Transcription Factor 7 | Metastasis | Inhibits metastasis | Chen et al. 2015 |
| TGFΒR2 | TGF Beta Receptor 2 | EMT | Inhibits EMT | Schultz et al. 2011 |
| TGIF2 | Transforming Growth Interaction Factor 2 | Epigenetics | Inhibits cell growth and differentiation, and induces apoptosis | Wu et al. 2016 |
| TPD52 | Tumor Protein D52 | Invasion | Inhibits cell migration and invasion | Kaller et al. 2011; Li et al. 2016 |
| TRAFD1 | Traf-Type Zinc Finger Domain Containing 1 | Immunity | Modulates T cell regulatory networks | Hart et al. 2019 |
| TREM2 | Triggering Receptor Expressed in Myeloid Cells 2 | Immunity | Represses phagocytosis | Alexandrov et al. 2013; Bhattacharje et al. 2016 |
| TWIST1 | Twist-Related Protein 1 | EMT | Inhibits EMT | Imani et al. 2017 |
| ULBP2 | UL16 Binding Protein 2 | Immunity | Suppresses NK immune surveillance | Heinemann et al. 2012 |
| WNT1 | Wingless-Related MMTV Integration Site | Stemness | Inhibits Wnt signaling | Hashimi et al. 2009 |
| WNT3 | Wingless-Related MMTV Integration Site | Stemness | Inhibits Wnt signaling | Kim et al. 2011 |
| YY1 | Yingyang1 Transcription Factor | Epigenetics | Inhibits tumor growth, cell proliferation, and differentiation | Chen et al. 2011; Kaller et al. 2011 |

A direct regulation of these genes by miR-34a using reporter assays has been validated.
