# Supplementary material for: MicroRNA-34a: Potent Tumor Suppressor, Cancer Stem Cell Inhibitor, and Potential Anticancer Therapeutic
Source: Front Cell Dev Biol. 2021 Mar 8;9:640587. doi: 10.3389/fcell.2021.640587 (PMC7982597; doi:10.3389/fcell.2021.640587)
Supplement: Supplementary file 2 [file Table_2.docx]

**Supplementary Table 2.** Reported functions of miR-34a in other cancers (as of Nov. 2020).

| **Cancer type** | **Reported functions (effects) of miR-34a** |
| --- | --- |
| **Pancreatic cancer** | Delivery of miR-34a decreased in vivo stemness markers SIRT1, CD44 and ALDH. (Pramanik et al., 2011) |
|  | Restoration of miR-34a in pancreatic CSCs significantly reduced in vitro cell proliferation, cell cycle progression, self-renewal, migration, and invasion. And it caused an inhibition in the mRNA expression of all the components of Notch pathway- Notch1, Notch 3 and its ligand Jagged1 and Hes1. (Nalls et al., 2011) |
| **Head and neck cancer** | Loss of TP53 leads to adrenergic trans-differentiation of tumor associated sensory nerves through loss of miR-34a, which increases cancer–nerve crosstalk and thereby promotes tumor progression. (Amit et al., 2020) |
| **Osteosarcoma (OS)** | miR-34a could restrain OS dedifferentiation into cancer stem-like cells by targeting plasminogen activator inhibitor-1 (PAI-1) and downregulating SRY-related-HMG box (SOX) 2. (Zhang et al., 2018) |
|  | miR-34a-5p promoted the multi-drug resistance of osteosarcoma by targeting the CD117/c-Kit gene (Pu et al., 2016) |
| **Gastric Cancer** | miR-34a negatively regulated stem cell-like characteristics by negative feedback regulation of Bmi-1 in gastric cancer. (Wang et al., 2016b) |
|  | Systemic delivery of miR-34a attenuated proliferation and migration of gastric CSCs by repressing the expression of CD44, decreasing levels of Bcl-2, Oct4, and Nanog genes and reducing 3D spheroid formations. (Jang et al., 2016) |
| **Hepatocellular Carcinoma (HCC)** | CD44 3' UTR functioned as a competing endogenous RNA (ceRNA) for competing miR-34a to enhance NK sensitivity of liver cancer stem cell by regulating ULBP2 expression in HCC. (Weng et al., 2019a) |
| **Urothelial Bladder Cancer (UBC)** | miR34a inhibited UBC stemness and chemoresistance in vitro and in vivo by directly targeting GOLPH3. miR-34a treatment decreased the sphere formation ability and the CD44+ cell population. (Zhang et al., 2017) |
|  | miR-34a was shown to significantly suppress CD44 expression, repress cell growth and invasion capability, and reduce chemoresistance. However, c-Myc acts as a ceRNA to sponge mir-34a, leading to the attenuation of the function of miR-34a in controlling CSC phenotypes in urothelial carcinoma. (Chen et al., 2019b) |
| **Mesothelioma** | c-Met, a direct target of miR-34a, was required for tumor migration/invasiveness and maintenance of the CSC population in malignant mesothelioma. (Menges et al., 2014) |
