# Supplementary material for: MicroRNA-34a: Potent Tumor Suppressor, Cancer Stem Cell Inhibitor, and Potential Anticancer Therapeutic
Source: Front Cell Dev Biol. 2021 Mar 8;9:640587. doi: 10.3389/fcell.2021.640587 (PMC7982597; doi:10.3389/fcell.2021.640587)
Supplement: Supplementary file 3 [file Table_3.docx]

**Supplementary Table 3.** The list of reported ceRNAs that act as miR-34a sponge (as of Nov. 2020).

| **ceRNA** | **Type** | **Regulated gene** | **Reference** |
| --- | --- | --- | --- |
| CircBIRC6 | Circular RNA | OCT4 and NANOG | Yu et al. 2017 |
| CircGFRA1 | Circular RNA | GFRA1 | He et al. 2017 |
| CircNFIX | Circular RNA | Notch1 | Xu et al. 2018 |
| Circ-DB | Circular RNA | Met | Li et al. 2018a |
| CircRNA-ASH2L | Circular RNA | Notch1 | Chen et al. 2019a |
| Circ_0009910 | Circular RNA | ULK1 | Cao et al. 2020 |
| CircINSR | Circular RNA | Bcl-2 and CyclinE2 | Shen et al. 2020 |
| TUG1 | LncRNA | VEGFA | Dong et al. 2016 |
| XIST | LncRNA | E2F3, Met, WNT1 | Song et al. 2016 |
| CCAT2 | LncRNA | FOXM1 | Chen et al. 2017 |
| HNF1A-AS1 | LncRNA | SIRT1 | Fang et al. 2017 |
| LINC00473 | LncRNA | ILF2 | Shi et al. 2017 |
| MUF | LncRNA | Snail1 | Yan et al. 2017 |
| NEAT1 | LncRNA | c-Met, Sirt1, Satb1, ACSL4 | Liu et al. 2017; Ding et al. 2017 |
| ANRIL | LncRNA | SIRT1 | Dong et al. 2018 |
| Lnc015192 | LncRNA | Adam12 | Huang et al. 2018 |
| SNHG7 | LncRNA | GALNT7, Notch1 | Li et al. 2018b |
| UFC1 | LncRNA | FOXP3 | Cao et al. 2015; Xi et al. 2018 |
| DANCR | LncRNA | JAG1 | Ma et al. 2019 |
| KCNQ1OT1 | LncRNA | ATG4B | Li et al. 2019a |
| LINC00346 | LncRNA | CD44, NOTCH1, and AXL | Xu et al. 2019 |
| MALAT1 | LncRNA | c-Myc, Met | Duan et al. 2019; Li et al. 2019b |
| RMRP | LncRNA | c-Myc | Xiao et al. 2019 |
| SNHG14 | LncRNA | HMGB1 | Jiao et al. 2019 |
| FEZF1-AS1 | LncRNA | Notch1 | Huang et al. 2020 |
| GCMA | LncRNA | Slug, Snail | Tian et al. 2020 |
| HCG18 | LncRNA | HMMR | Li et al. 2020 |
| MIR31HG | LncRNA | c-Met | Chu et al. 2020 |
| ASS1P3 | Pseudogene | ASS1 | Wang et al. 2019 |
| LDHA | The protein coding gene | PDL1 | Huang et al. 2017 |
| PDL1 | The protein coding gene | LDHA | Huang et al. 2017 |
| CD44 | The protein coding gene | ULBP2 | Weng et al. 2019 |
| c-Myc | The protein coding gene | CD44 | Chen et al. 2019b |
